# Unexpected Reactivity of Zirconium Carbide on Nickel/Yttria-Stabilized Zirconia Boosts CO_2_/H_2_O Co-electrolysis to Methane

**DOI:** 10.1021/acs.chemmater.6c00480

**Published:** 2026-04-30

**Authors:** Christoph W. Thurner, Kevin Ploner, Patrick Obendorf, Daniel Winkler, Alexander Genest, Günther Rupprechter, Simon Penner, Bernhard Klötzer

**Affiliations:** † Institute of Physical Chemistry, 27255 University of Innsbruck, Innrain 52c, 6020 Innsbruck, Austria; ‡ 256221 Plansee SE, Metallwerk-Plansee-Straße 71, 6600 Reutte, Austria; § Institute of Materials Chemistry, Technische Universität Wien, Getreidemarkt 9/BC/01, 1060 Vienna, Austria; ∥ Ceratizit S.A., Rue de Holzem 101, 8232 Mamer, Luxembourg

## Abstract

The high-temperature co-electrolysis of CO_2_ and H_2_O in solid oxide cells is a promising avenue toward renewable energy storage and climate change mitigation. The present study identifies fundamental electrochemical pathways toward methane under syngas-rich electrochemical conditions and differentiates the specific reactivities of the observed carbon deposits. A thin-film electrode comprising Ni on yttria-stabilized zirconia (YSZ) was evaluated by operando near-ambient pressure X-ray photoelectron spectroscopy (NAP-XPS) and mass-spectrometric product detection under strongly cathodic electrolysis conditions with predominantly CO and H_2_ in the gas phase. Under these conditions, the reduction of CO toward elemental carbon species takes place. A hitherto unknown methane-forming mechanism via polarization-induced carbon spillover from Ni to YSZ was identified, causing ZrC_*x*_ formation at the electrolyte surface near the Ni/YSZ boundary. Hydrolysis of this ZrC_*x*_ species by added traces of steam enhances the methane selectivity. Our experimental results, corroborated by density functional theory (DFT) modeling, enable better control of high-temperature CO_2_–H_2_O co-electrolysis for product-selective renewable energy storage.

## Graphical abstract

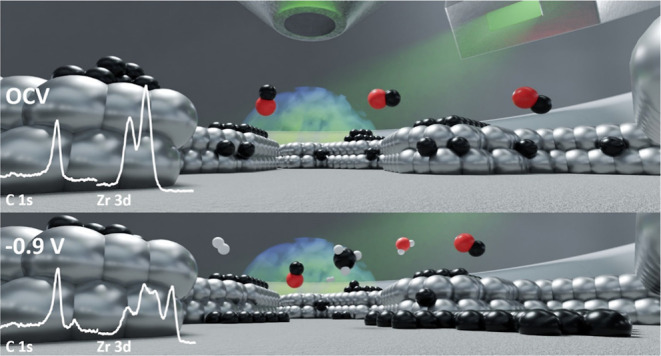

## Introduction

High-temperature co-electrolysis of CO_2_ and H_2_O in solid oxide cells contributes to climate change mitigation by producing energy carriers like CO and H_2_, while storing renewable electric energy.^1–4^ As depicted in Figure 1, CH_4_ can be formed thermochemically via (co)­generated H_2_ reacting with CO (R6) or CO_2_ (R5) in a separate reactor.^1,5^ Avoiding these additional Sabatier-type steps, direct CH_4_ formation via electrochemical reaction R1 ^4^ enables even further energy storage through elemental carbon formation (R3).

R1 $${CO}_{2}+{2H}_{2}O⇌{CH}_{4}+{2O}_{2}$$

**Figure 1:**
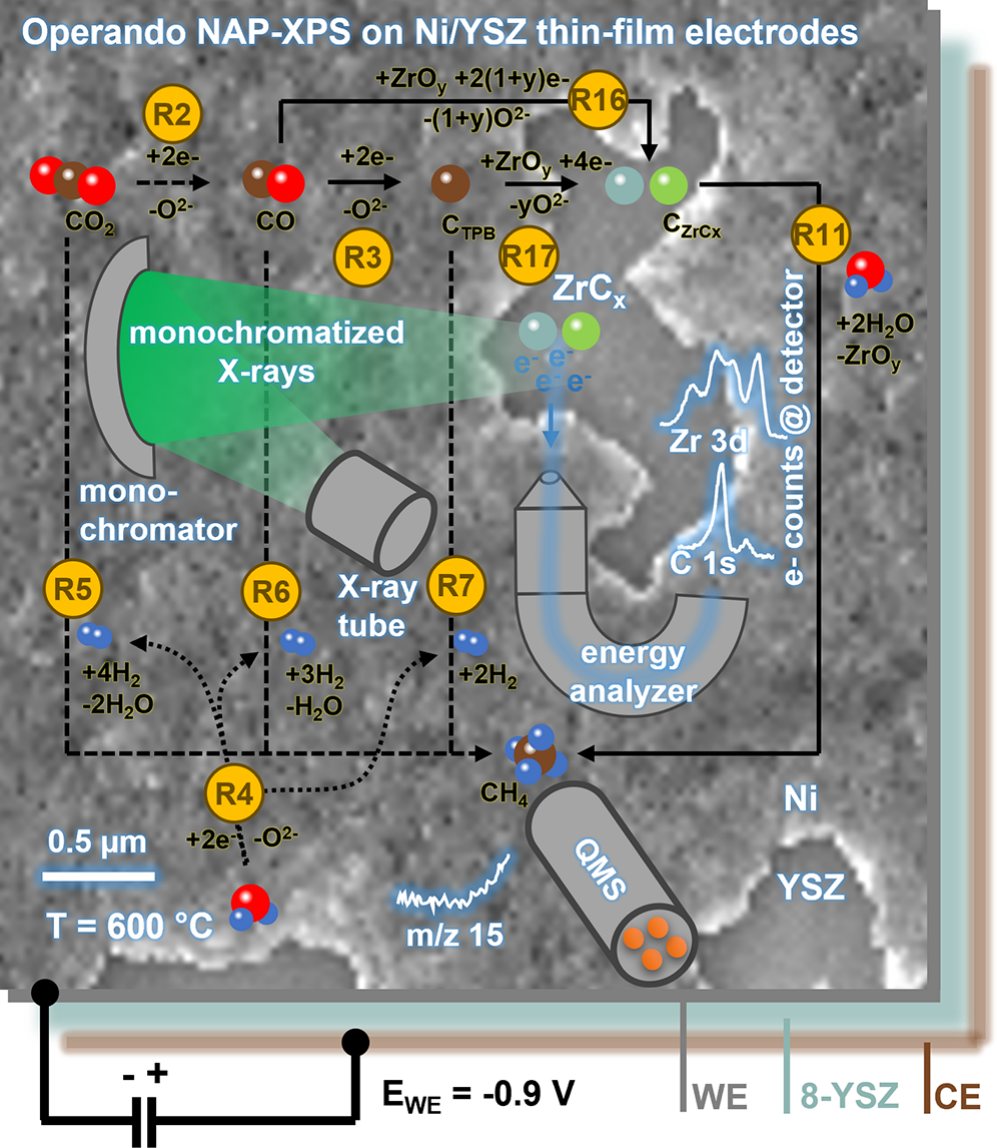
Operando near-ambient pressure X-ray photoelectron spectroscopy (NAP-XPS) approach is illustrated next to the methane-generating reaction network in high-temperature CO_2_/H_2_O co-electrolysis on electrified thin-film Ni/8-YSZ interfaces. The thin-film working electrode (WE) mimics key electrochemical features of a commercial 3D-porous Ni/8-YSZ bulk cermet, while allowing detection of photoelectrons escaping from the electrochemically active interfaces. The network involves electrochemical formation of intermediates (R2–R4) and thermochemical hydrogenation (R5–R7) to methane. R16 and R17 describe ZrC_*x*_ formation via electrochemical ZrO_*y*_ reduction and reaction with CO or C. The resulting ZrC_*x*_ on the yttria-stabilized zirconia (YSZ) domains is detected by energy-resolved photoelectron counting, triggered by monochromatized X-rays irradiating the polarized Ni/YSZ interface. Hydrolysis of ZrC_*x*_ to CH_4_ (R11) was tracked via online QMS monitoring *m*/*z* = 15. The thin-film cell remained isothermal at 600 °C throughout all experiments. The scanning electron microscopy (SEM) and energy-dispersive X-ray spectroscopy (EDXS) results of the as-prepared 2D-holey Ni thin-film WE, showing local Ni dewetting and Ni/8-YSZ phase boundary formation, and a schematic cross-section of the electrochemical setup used in the NAP-XPS chamber are illustrated in Section S1.

Some of the potentially involved reaction steps in the overall reaction R1 shown below represent electrochemical reactions (R2, R3, and R4), whereas others are purely thermo-catalytic ones (R5, R6, and R7).^5^

R2 $${CO}_{2}+{2e}^{-}⇌CO+O^{2-}$$

R3 $$CO+{2e}^{-}⇌C+O^{2-}$$

R4 $$H_{2}O+{2e}^{-}⇌H_{2}+O^{2-}$$

R5 $${CO}_{2}+{4H}_{2}⇌{CH}_{4}+{2H}_{2}O$$

R6 $$CO+{3H}_{2}⇌{CH}_{4}+H_{2}O$$

R7 $$C+{2H}_{2}⇌{CH}_{4}$$

The formation of methane stems from the reaction of CO_2_, CO, or C with (co)­generated hydrogen originating from electrochemical water splitting. In the conventional methanation reactions R5 and R6, two mechanistic scenarios play a central role on supported Ni catalysts: (a) a “low-temperature” hydrogen-assisted CO_2_/CO dissociation step via the key intermediates HCOO_ads_ or CHO_ads_, respectively,^6–8^ and (b) a “high-temperature” direct dissociative mechanism transforming CO_2_/CO into intermediate C_ads_, followed by the reaction of the latter with H_ads_ to CH_4_.^6,7^ Beside temperature, the predominant mechanistic pathway depends on the H_2_ to CO_2_/CO ratio, possible side reactions, and the vacancy concentration in the ZrO_*x*_ support, which in turn depends on the reductive gas-phase potential.^6–8^

Electrifying the interface of a Ni^0^/8 mol % yttria-stabilized zirconia (8-YSZ) electrode supplements the prevailing thermo-catalytic reaction pathways by generating additional carbon via R2 and R3 electrochemically. The additionally formed key intermediate C_ads_ can then undergo subsequent hydrogenation via R7.^6,9^ Thereby, the kinetics of the initial elementary step C_ads_ + H_ads_ → CH_ads_ depends on the C_ads_ coverage^7^ and may be promoted electrochemically.

In essence, on (supported) Ni catalysts at temperatures <500 °C, the entire hydrogen-based reaction network toward CH_4_ (R5–R7) is mechanistically linked to a stepwise hydrogenation of key intermediate C_ads_,^6–9^ whose coverage appears electrochemically tunable via R3, supplementing the prevailing thermo-catalytic reaction pathway kinetically.^7^

In principle, the electrochemical reaction products from R2 (CO) and R3 (C) can be generated thermo-catalytically, as well, via the water–gas-shift equilibrium (R8), and the Boudouard equilibrium (R9) or CO reduction by H_2_ (R10).^5,7^

R8 $${CO}_{2}+H_{2}⇌CO+H_{2}O$$

R9 $$2CO⇌C+{CO}_{2}$$

R10 $$CO+H_{2}⇌C+H_{2}O$$

However, due to the near-ambient pressure (NAP)-XPS-specific experimental pressure and temperature conditions in this work, the competing carbon-generating forward reactions R9 and R10 are considered negligible.^5,10^

As CO, C, and H_2_ (R2–R4 in Figure 1) can approach high quantities at sufficiently high cathodic current densities, a sequence of R3 followed by R7 has been proposed to explain CH_4_ formation under strongly reducing co-electrolysis conditions on Ni/8 mol % yttria-stabilized zirconia (8-YSZ) cermet electrodes.^2,11^

However, reactions involving C_ads_ species can also lead to surface coking and Ni/YSZ triple phase boundary (TPB) blocking.^12^ As direct methane formation under H_2_O/CO_2_ co-electrolysis conditions is definitely sustainable,^4,11,13^ irreversible blocking of the entire Ni surface—including the TPB-near regions—by unreactive carbon can be excluded and alternative carbon-depleting and/or -utilizing pathways toward CH_4_ must be considered.

Generally, the inherent activity of distinct material- and site-specific forms of carbon appears worthwhile to be tested for effective decoking and hydrocarbon formation. Distinguishing reactive from inert carbon species^14^ is key for knowledge-based steering of desired reaction channels—a factor relevant also to direct methane solid oxide fuel cells (SOFCs)^15^ in reversible storage/release scenarios.^4^

The known carbon mobility in nickel^16^ and its recently reported spillover to zirconia^17^ led us to consider an additional and mechanistically so far unexpected possibility, namely the hydrolysis of intermediate carbide species,^18–20^ particularly of ZrC_*x*_

R11 $$ZrC+{2H}_{2}O⇌{CH}_{4}+{ZrO}_{2}$$

R12 $$ZrC+{3H}_{2}O⇌CO+{3H}_{2}+{ZrO}_{2}$$

R13 $$ZrC+{4H}_{2}O⇌{CO}_{2}+{4H}_{2}+{ZrO}_{2}$$

These reactions are plausible under typical co-electrolysis conditions where trace H_2_O is always present. The passivation of zirconium carbide under moist environments^21^ implies that its bulk carbide-terminated surface reacts efficiently with H_2_O, forming a thin protective ZrO_2_ layer.^22^ Beside others,^19^ titanium carbide also shows CH_4_ formation under hydrolysis conditions.^18^

To probe the specific reactivities of variable carbonaceous species on electrified Ni/8-YSZ interfaces under near-operational conditions, we used our custom-designed thin-film electrode approach^23^ combined with operando NAP-XPS with synchronous electrochemical activity control and quadrupole mass spectrometric (QMS) product detection^24^ (Figure 1 and Section S1).

The NAP-XPS conditions favor controlled carbon formation^5,10^ via reaction R3 only, while suppressing competing carbon-generating pathways (i.e., R8 due to avoiding CO_2_, R9 and R10 due to pressure and temperature conditions), leading to electrochemically induced carbon species through field-driven CO activation near the TPB.^25^ The terms “C_TPB_” and “C_ZrCx_” refer here to XPS-detectable carbon species accumulating on Ni or YSZ surfaces as a result of TBP electrification. To elucidate the mechanism of initial C atom formation from CO, density functional theory (DFT) calculations were employed.

The study proceeds by examining

(a) The electrochemical formation, surface accumulation, and dissolution of C_TPB_ on Ni (R3), as well as the (re)­formation of polarization-induced C_ZrCx_ (R16, R17), both experimentally and theoretically,
(b) the effect of *H*_2_(*g*) on C_TPB_ (R7) and C_ZrCx_ deposits, and ZrC_*x*_-mediated C*H*_4_(*g*) formation in the presence of H_2_O­(g) (R11).

In extended 3D-porous cermet electrodes, the electrochemically active zone is in close proximity to the dense solid electrolyte and remains spectroscopically inaccessible to surface-sensitive techniques such as NAP-XPS. To overcome this inherent limitation and enable direct spectroscopic access to the relevant electrochemical processes, we deliberately employed a quasi-2D-structured porous model electrode architecture as a model system for mechanistic investigations, with the drawback of neglecting gas distribution effects usually present in 3D porous structures.

However, only this methodologically tailor-made approach based on operando NAP-XPS allowed us to demonstrate evidence for an additional unrecognized CH_4_-forming reaction mechanism active under strongly cathodic co-electrolysis conditions.

## Experimental Section

### Electrolysis Cell Preparation

For a detailed description of the preparation of the button cells (Ø 5 mm), we refer to a recent publication.^23^ This cell is layered as follows (from counter electrode (CE) side to working electrode (WE) side): porous Pt-current collector (≈5 μm)//porous Pt/Gd_0.1_Ce_0.9_O_1.95_ (GDC-10; ≈10 μm)//GDC-10 adhesion-promoting layer (≈5 μm)//polycrystalline (ZrO_2_)_0.92_(Y_2_O_3_)_0.08_ (8-YSZ) electrolyte substrate (150 μm)//Ni thin-film 2D-network (100 nm). On the commercially available 8-YSZ solid electrolyte and GDC-10 adhesion layer (Kerafol, Germany), the CE was spin coated by means of a Pt/GDC-10 cermet ink (volume ratio 52:48) and then subsequently paint brushed with a Pt-paste (Tanaka Kikinzoku Kogyo K.K., Tokyo, Japan). After being sintered at 1150 °C in air for 3 h, the fabricated porous CE combines an optimized number of TPB sites and a high surface area, resulting in superior electrocatalytic activity. This well-established CE design enables negligible overpotentials with respect to thin-film based WEs.^26^ The WE thin film was prepared by physical vapor deposition. Ni (99.9% purity) was thermally evaporated in high vacuum (background pressure ≈1 × 10^–7^ mbar), coating the preheated (330 °C) 8-YSZ substrate with a deposition rate of ≈2 nm/min. After annealing in 50 mbar H_2_ and 950 mbar He atmosphere in a quartz tube furnace at 800 °C, local dewetting of the 8-YSZ substrate leads to an electronically conducting, cermet-like metallic 2D-network (Figure S1A).

### CE Properties and Setup

The choice of the CE is related to the specific cermet properties of Pt and 10 mol % gadolinium-doped ceria (GDC-10). In essence, the superior electrocatalytic activity of Pt is associated with H_2_ activation, while GDC-10 provides excellent oxygen exchange kinetics, leading to a negligible voltage-drop of the CE relative to thin-film WEs.^26^ As CO electrolysis via R3 leads to surface carbon species under cathodic conditions at the WE only, the CE activity is restricted to the anodic oxidation reactions of CO or H_2_ and separated electrode compartments are not mandatory for the operando detection of gaseous products formed at the WE.^27^ Nonetheless, the thermo-catalytic CO methanation reaction rate (R6) of Pt is much smaller than that of Ni under equal conditions.^28^ Consequently, the chosen cell design and intrinsic material properties enable to sensitively distinguish between electro- and thermo-catalytic methane formation on the WE only, while operating the cell stack in a single atmosphere^29^ in a specially designed NAP-XPS chamber.^24^

### WE Characterization

The WE thin-film was characterized by scanning electron microscopy (SEM). Analysis was done by utilizing a FEI Quanta 650 FEG, operating under an electron acceleration voltage of 15 kV under high vacuum (5 × 10^–6^ mbar). Elemental information was obtained by energy-dispersive X-ray spectroscopy (EDXS) mapping (Figure S1A). For topological data recorded by atomic force microscopy (AFM), we refer to ref 23.

### Operando NAP-XPS

For electron-spectroscopic operando investigations under electrochemical reaction conditions, we carried out experiments using a custom-designed laboratory-based NAP-XPS system from SPECS. A comprehensive description of the entire system can be found in ref 24. In summary, the core XPS setup consists of an μFOCUS 600 NAP monochromatic small spot Al Kα X-ray source (spot size of 300 μm) and a hemispherical energy analyzer (SPECS Phoibos 150 NAP) equipped with a 1D delay line detector. This setup is configured vertically within a μ-metal analysis chamber, which shields the system from external electric and magnetic fields. To enable XPS experiments in variable gas environments, a differentially pumped electrostatic lens system separates gas molecules from the photoelectrons directed toward the analyzer. This allows XPS experiments to be conducted while the analysis chamber is backfilled with gases or gas mixtures up to a pressure of 25 mbar, supplied via mass flow controllers (Bronkhorst). For real-time monitoring of gas phase composition and surface product formation during in situ XPS observations, two quadrupole mass spectrometers (QMS)—Hiden HPR-40 and MKS Instruments e-vision2—are installed in the first and second differential pumping stages beyond the analyzer nozzle, respectively. These mass spectrometers are operated according to specific sensitivity requirements. Gases generated at the sample surface within the chamber are pumped through the nozzle and can be promptly detected, providing real-time information on product formation.^23^ As indicated in Figure S1B, an IR laser with a maximum power of 120 W is mounted underneath the analysis chamber. It allows precise heating of samples from the rear through an 8 mm hole in the sample holder. Temperature measurements are facilitated by a K-type Ni/NiCr thermocouple placed in the vicinity of the surface region observed by XPS. To ensure uniform heat distribution across the WE and, consequently, uniform WE surface temperature, the thermocouple is attached to an uncoated region of the pristine YSZ disc. This attachment is achieved by sintering the wires onto the YSZ surface by using a Ni-paste droplet (Figure S1B). For a more detailed description of the custom-made sample holder designed specifically for solid-state electrochemical studies, we refer to ref 24. For the electrochemical polarization of the cell, a Biologic (SP-200) potentiostat is connected to the sample holder stage, allowing for potentiostatic and galvanostatic, as well as potential sweep experiments.

Due to issues related to the placement of a reference electrode on a solid electrolyte, a two-electrode configuration was used. To prevent ground-loop contributions, the operating mode was set to “floated”, which means that the potentiostat was grounded directly to the XPS analyzer. The WE was connected to ground in all experiments, meaning that the Ni network aligns to the Fermi level of the analyzer. Although the ground contact senses the current, this connection is reasonable, as contributions from the photoemission current were detected to be in the low nano-ampere regime, representing a negligible contribution to the measured current. For electrochemical polarization, a DC voltage (*E*_WE_) was set between the CE and WE (ground). For electrochemical data analysis, the associated software program EC-Lab v 11.43 was utilized. To carry out XPS measurements, the X-ray source was set to 70 W and 13 kV. For all recorded spectra, the analyzer was adjusted to a pass energy of 50 eV. XPS data analysis is based on the CasaXPS software, version 2.3.24 PR1.0 (Casa Software Ltd.).^30^ For background correction, a Shirley background was applied to the C 1s, O 1s, and Zr 3d spectra. The Ni 2p_3/2_ peak can be associated with a binding energy (BE) of 852.8 eV for the metallic component,^31^ while the BE of the Zr 3d_5/2_ peak is close to 183.0 eV, indicating the exclusive presence of Zr in a Zr^4+^O_2_ environment.^32^ The analysis of components within the C 1s region is based on a routine established by Biesinger.^33^

### Computational Details

DFT calculations were carried out using the PBE^34^ functional in the plane-wave-based program package VASP^35^ including the projector augmented-wave approach.^36^ An energy cutoff of 400 eV was used together with a 1 × 1 × 1 grid with a Gaussian smearing (sigma = 0.05 eV). Structures were considered optimized when the gradients dropped below 0.05 eV/Å. Barriers were estimated using the nudged elastic band algorithm with 5 images.^37^

The sizes of these models for DFT calculations (8 Y, 40 Zr, 92 O atoms) are the range of previous models^38^ regarding the particle size as well as ZrO_2_. We decided to use finite Ni particles because one of the aims of the present work was to study whether a saturation with carbon may be reached. A Ni_7_ particle was adsorbed at one of the vacancies in the top layer, with the motivation of having a small terrace available consisting of 6 Ni atoms with a single Ni atom on top of this particle to facilitate CO activation, inspired by a work on CO on Pt.^39^ A second model was created to understand the supersaturation of the particle with carbon using a Ni_4_ tetrahedron.

## Results and Discussion

### Electrochemical Formation, Accumulation, and Dissolution of C_TPB_ in the Presence of Pure CO Atmosphere

In pure CO, C_TPB_ can be grown electrochemically via reaction R3 (600 °C, 0.5 mbar, *E*_WE_ = −0.9 V), as detailed in Section S2, and is characterized by two C 1s components at binding energies (BEs) of 284.6 and 285.0 eV. However, under unpolarized conditions (over ≈100 min) and stepwise cathodic polarization increase (i.e., −0.1 V/step kept constant over 10 min each step) until −0.8 V no carbon deposition in CO­(g) was observed (Section S2). According to Biesinger et al.,^33^ graphitic-like carbon consists of sp^2^-bonded carbon at 284.5 eV and a minor sp^3^ component at 285.0 eV, respectively. Thus, C_TPB_ initially corresponds to C_sp_3__, transitioning upon accumulation over time to predominantly C_sp_2__. The related Section S3 reveals that the Zr 3d integral peak area remains basically constant during growth, while carbon accumulation coincides with a strong decrease of the Ni 2p signal, indicating preferential C_TPB_ deposition on metallic Ni. Repeated current–voltage cycles between 0 and −0.9 V suggest a gradual decrease in the attainable faradaic cathodic current due to progressive blocking of C_TPB_-forming sites at the Ni/8-YSZ boundary (Section S4). In any case, the current response exhibits also a superimposed nonfaradaic—most likely ohmic, contribution of the strongly reduced 8-YSZ electrolyte surface (Section S5).

Optical evidence for progressive C_TPB_ accumulation under a cathodic bias is presented in Section S6, showing increasingly carbon-darkened regions that fade over time after switching to the open circuit potential (OCV). This fading is attributed to enhanced solubility of segregated carbon into the Ni bulk, along with carbon clean–off reactions. To avoid the latter, carbon dissolution kinetics were studied under high vacuum (HV), with Section S7 highlighting remarkable differences between a pristine sample (S2-d1) and a carbon presaturated one (S1-d3), which had undergone three prior carbon growth cycles.

### Polarization-Induced C_ZrCx_ Formation in a Pure CO Atmosphere

As a first step, the carbon growth/vacuum dissolution experiment on a pristine Ni/YSZ electrode (S2-g1 in Figure S3B and S2-d1 in Figure S7) results in pronounced C_TPB_ growth on the Ni domains and moderately carbon presaturated Ni bulk. C_ZrCx_ formation could not be initiated on any pristine electrode and required preceding electrochemical C_TPB_ growth. Once sample S2 was presaturated with C, each subsequent switch to cathodic conditions (g2, g3 in Figure 2A) induced C_ZrCx_ formation in CO­(g).

**Figure 2:**
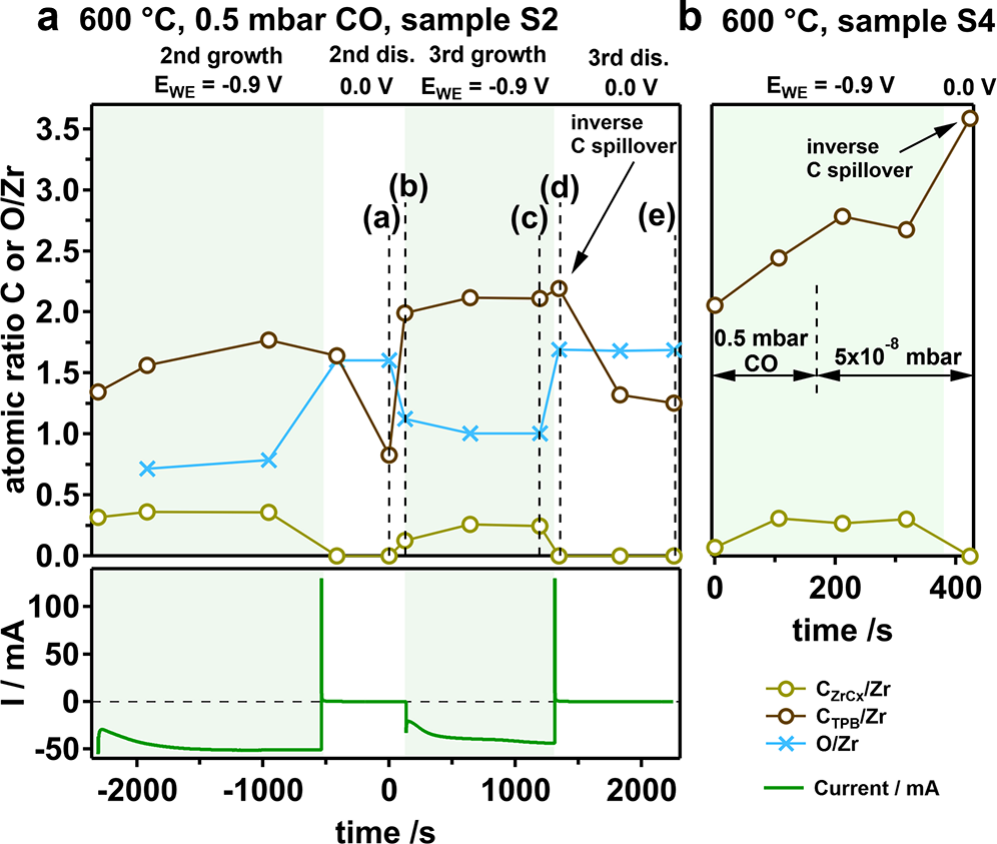
(A) Time- and polarization-dependence of the indicated ratios of the integrated XP spectral intensities (*E*_WE_ = −0.9 V within the green-shaded areas g2 (2nd growth) and g3 (3rd growth)): C_ZrCx_/Zr (light green), C_TPB_/Zr (brown) and O/Zr (light blue). The respective current vs time data are plotted in the bottom panel of A (dark green trace). (B) Indicated ratios of the integrated XP spectral intensities (C_ZrCx_/Zr: light green, C_TPB_/Zr: brown) recorded while switching from CO atmosphere to vacuum conditions under polarization (*E*_WE_ = −0.9 V within the green-shaded area), and returning to OCV conditions.

To gain a more detailed understanding of the ZrC_*x*_ formation mechanism, the calculated atomic ratios of C_TPB_/Zr, C_ZrCx_/Zr, and O/Zr were evaluated as a function of time, while the voltage was switched between *E*_WE_ = −0.9 and 0.0 V (Figure 2A). In this context, the ratio of C_TPB_/Zr was calculated to highlight changes of the total C_TPB_ coverage (located exclusively at the Ni surface, and consisting mainly of C_sp_2__), whereby the largely graphite-free YSZ surface, represented by the entire integrated Zr 3d intensity, served as a reference. Equally, the C_ZrCx_/Zr ratio should provide information about potential changes in the carbide species normalized to the Zr reference. Determining the ratio O/Zr provides an indicator for oxygen depletion of the YSZ surface.

During the second cathodic polarization period (green shaded area, −2500 to −500 s, g2), a stable C_ZrCx_/Zr ratio (light green trace) persists. At the same time, the C_TPB_/Zr ratio (brown trace) increases, suggesting active growth of additional electrochemically induced C_TPB_ via R3. Because of the simultaneous formation of ZrC_*x*_, oxygen ions are replaced on the YSZ surface, and the ratio O/Zr (light blue trace) is thus stabilized at a low level.

As the preceding rapid drop of the C_TPB_/Zr ratio between −400 and 0 s (1.64 to 0.82) occurs upon depolarization in CO­(g), it is expected that C*O*_2_(*g*)—generated during cathodic polarization on the CE (anode)—contributes to the overall loss of carbon intensity via the reverse reaction R9 (2CO ⇌ C + CO_2_), in addition to ongoing but rather slow C dissolution anticipated on a carbon presaturated Ni film (cf. Sections S7 and S8).

Application of 0.0 V conditions causes the carbide to vanish instantaneously, and the ratio C_TPB_/Zr (brown trace) starts to decrease because of ongoing C dissolution in the Ni bulk and is supplemented by slow carbon clean–off reactions. As ZrC_*x*_ is a polarization-stabilized species with Zr centers close to the Zr^0^ state, the injected electrons required for the reduction of Zr^4+^ to this reduced state are stored in the formed ZrC_*x*_ phase. Upon switching to 0.0 V, a reversible discharge to oxygen-coordinated, cationic Zr takes place, resulting in a pronounced current spike (Figure 2A, green current trace). In summary, switching between 0.0 V and −0.9 V revealed the partially reversible discharge current characteristics of the cell.

During the third cathodic polarization period (100 to 1200 s, g3), the intensity trends are qualitatively quite similar to the second one, but the ratio C_TPB_/Zr (brown trace) reaches even higher values, indicating progressive accumulation of carbon. After switching back to 0.0 V, the C_TPB_ component initially increases, which suggests a potential “inverse” spillover effect of C_ZrCx_ on YSZ back to C_TPB_ on Ni. XP spectra recorded before, during, and after the polarization-induced C_ZrCx_ formation (regions d2/g3/d3, indices (a–e)) are visualized in Figure 3.

**Figure 3:**
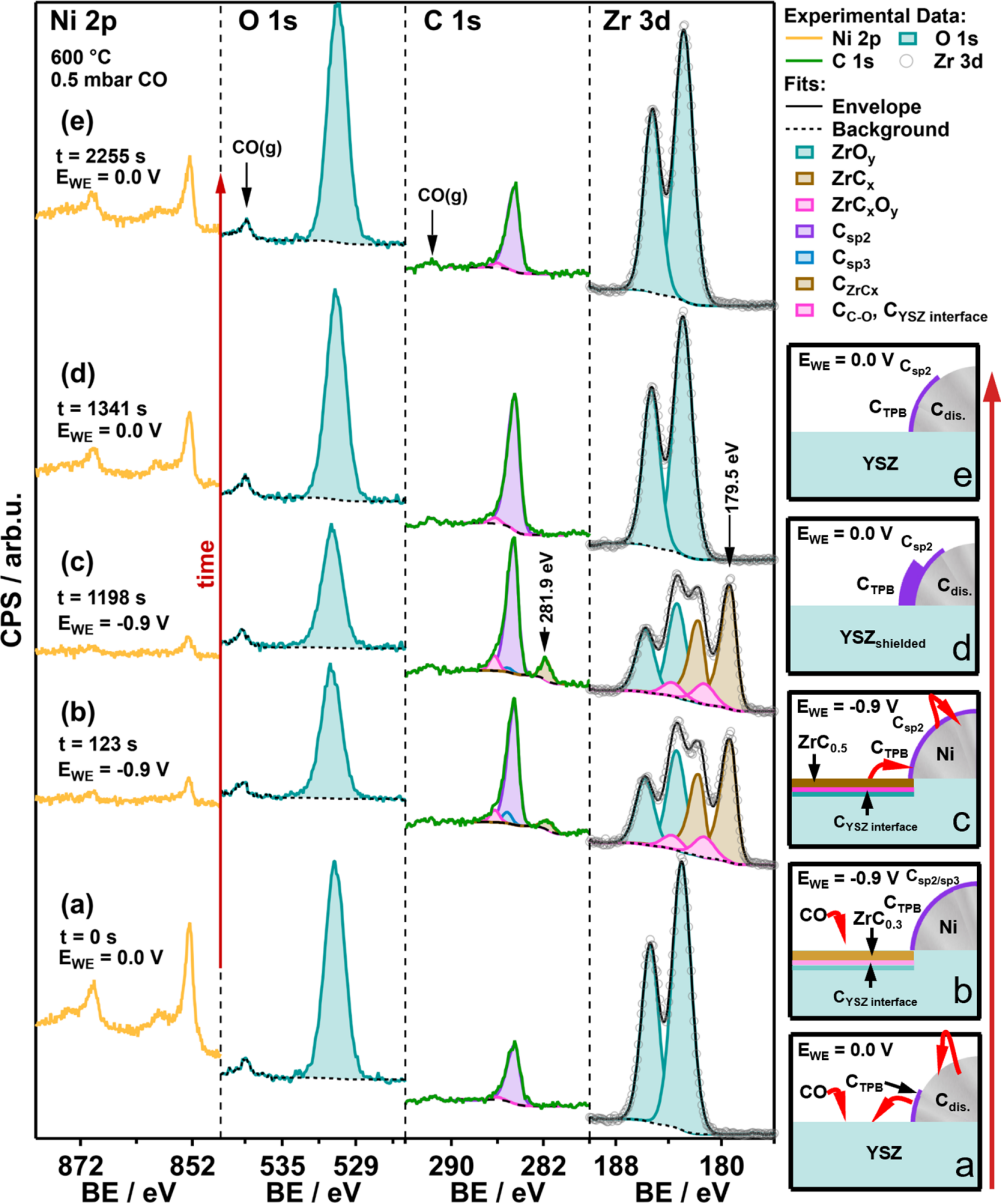
Formation of polarization-induced C_ZrCx_ in pure CO (0.5 mbar) is recorded by plotting the evolution of Ni 2p, O 1s, C 1s, and Zr 3d spectra with applied voltage (*E*_WE_) and time (bottom to top) at 600 °C. C 1s components: C_sp_2__ (violet, and main contributor to C_TPB_), C_sp_3__ (blue, from polarization-induced segregation of C_dissolved_ from Ni), C_C–O_/C_YSZ interface_ (pink, mainly at the YSZ/ZrCx interface), and C_ZrCx_ (brown, carbidic carbon). Zr 3d components: ZrO_*y*_ (light blue), ZrC_*x*_ (brown) and ZrC_*x*_O_*y*_ (pink). Sketches (a) to (e) schematically depict the Ni^0^/8-YSZ interface states, reflecting relative spectral intensity changes associated with electrochemical (de)­polarization. (a) Initial, depolarized, C-supersaturated state post two electrolysis cycles (g1/g2). (b) Cathodic polarization includes rapid carbide formation (≈123 s), extensively covering the electrolyte surface accompanied by the formation of oxycarbidic ZrC_*x*_O_*y*_ interface. Significantly decreased Ni 2p intensity indicates large-area Ni-shielding by fast surface carbon segregation from the C-supersaturated Ni film. (c) Moderate C_ZrCx_ growth at ≈281.9 eV coupled with increasing C_YSZ interface_ signals (≈286.3 eV), with a rather unchanged ZrC_*x*_/ZrC_*x*_O_*y*_ signal—indicating carbon enrichment (*x* = 0.3 → 0.5) of the ZrC_*x*_ layer. Simultaneously, C_sp_3__ (≈285.0 eV) vanishes, pointing to conversion into C_sp_2__. (d) Depolarization to 0.0 V partially restores the Ni signal (due to C redissolution) and triggers rapid decarburization, leading to YSZ-shielding carbon deposits near the TBP. (e): full recovery; residual C 1s stems from unreactive carbon persisting on Ni and decomposed carbon structures fully recovering the ZrO_*y*_ signal.

The experiment of Figure 2B was conducted to corroborate the suggested inverse carbon spillover effect. At first, both C_TPB_ and C_ZrCx_ were accumulated in a CO atmosphere by polarization (brown trace, until ≈175 s). Thereafter, CO was pumped off while maintaining cathodic conditions. Upon reaching 5 × 10^–8^ mbar base pressure, the ratio C_TPB_/Zr decreases, indicating some carbon bulk dissolution in Ni, but the C_ZrCx_/Zr ratio remains unchanged, indicating the stabilization of the carbide species by the applied electrical field. However, after switching to 0.0 V, the C_ZrCx_/Zr ratio drops immediately to zero, while the ratio C_TPB_/Zr shows a pronounced increase with time, thus proving that C_ZrCx_ stored in the ZrC_*x*_ phase can be effectively used to repopulate C_TPB_ on Ni via the suggested inverse carbon spillover process.

In Figure 3 spectra series (a), corresponding to *t* = 0 s in Figure 2, the sample exhibits an oxidic but strongly surface-reduced 8-YSZ state, concluded from the O/Zr ratio of 1.54, lower than the stoichiometric ratio of 1.84 and the 1.81 measured on the electrode just before g1 under 0.5 mbar of CO at 600 °C.

With respect to carbon solubility, it has been reported that Ni interacts with C at 600 °C, forming a solid solution (herein denoted C_dissolved_) in contact to surface species of segregated amorphous and graphitic carbon.^40^ A comparison of the BEs of Ni^0^ and graphitic C_sp_2__ vs Ni and C_dissolved_ suggests a partial charge of Ni^δ+^ and C^δ−^ in the latter phase, i.e., a slightly positively polarized Ni state induced by the more electronegative dissolved C atoms. Binding energies (BE) of the Ni 2p_3/2_ and C 1s signal of ≈853 eV^40,41^ and 283.3 eV^42^ were assigned to the bulk-located solid solution, whereas Ni^0^ and graphitic C_sp_2__ are referenced with 852.8 eV^31^ and 284.5 eV,^33^ respectively, whereby the latter is close to C_sp_2__ grown via CO electrolysis at 284.6 eV (Figure S9).

The assignment of partial charges rationalizes how the polarization-induced segregation of C_dissolved_ observed in (b) can shield Ni intensity, following a similar pattern as the previously reported temperature-induced segregation of carbon toward an initial disordered C_sp_3__ and C_sp_2__ containing layer (R14), which eventually ripens toward sp^2^-ordered carbon species^40^ (R15)­

R14 $${Ni}^{\delta +}+C_{dissolved}^{\delta -}⇌{Ni}^{0}+C_{{sp}_{3}/disordered}+C_{{sp}_{2}/disordered}$$

R15 $$C_{{sp}_{3}/disordered}+C_{{sp}_{2}/disordered}\to C_{{sp}_{2}/ordered}$$

Since C_sp_3__ can be distinguished from C_sp_2__ (Section S2), it serves as a marker to follow transition R15. A high-resolution representation of the fitting approach based on ref 33 is provided in Section S9. On this basis, we suggest that cathodic polarization leads to a sudden lowering of the solubility of C atoms in the Ni metal bulk by depleting Ni^δ+^ states via cathodic electron injection into the electrode film.

A strong ZrC_*x*_ contribution (49 atom %) in the Zr 3d region and the associated decrease of the O 1s intensity indicate large-scale YSZ surface transformation into a Zr carbide top layer. The rise in the correlated C_YSZ interface_ component points to “oxycarbidic” carbon at the ZrC_*x*_/ZrO_*y*_ interface and favors the picture of a rather thin, but surface-covering ZrC_*x*_ layer, whose ZrC_*x*_O_*y*_ interface exhibits intermediate Zr redox states (Section S10).

Ongoing carburization (coupled increase in C_ZrCx_ and C_YSZ interface_, while ZrC_*x*_ and ZrC_*x*_O_*y*_ remains constant) manifests itself in (c), along with gradual C_sp_3__ loss interpreted as transformation into C_sp_2__ via R15 (Section S9), as described in ref 40.

The observed extensive, widespread, and almost instantaneous electrolyte surface carburization suggests a highly effective carbon supply toward YSZ via the gas phase, beyond local TPB-near spillover of C_TPB_ residing at Ni to 8-YSZ. The latter is confirmed via partial ZrC_*x*_ formation from Ni-covering C_TPB_ under HV conditions upon polarization (cf. Section S11). As summarized in Section S12/Table S1, the degree of ZrC_*x*_ formation correlates with the CO partial pressure and is lowered under HV conditions, indicating that the indirect carbon supply route to the electrolyte is less efficient in the absence of CO­(g). Beyond the C_TPB_ → YSZ spillover pathway (cf. R17 in Figure 1), direct electrochemical CO activation at the negatively polarized and therefore partially oxygen-deficient, 8-YSZ electrolyte surface is proposed as an extension of R3

R16 $$xCO+{ZrO}_{y}+2\left(x+y\right)e^{-}⇌{ZrC}_{x}+\left(x+y\right)O^{2-}$$

We further suggest that R16 is self-inhibited in regions where the ZrC_*x*_ layer is already present due to locally blocked oxygen anion transport. Consequently, the prevalence of O^2–^ conduction toward the 8-YSZ bulk at the carbide-free YSZ surface area and the enhanced electronic conductivity of the ZrC_*x*_ layer—which shifts the electrical field gradient toward the newly formed YSZ/ZrC_*x*_ triple phase boundary—combine in favor of a lateral/front-like expansion of a thin ZrC_*x*_ layer.

The sharp rise in Ni 2p intensity in (d) reflects large-area decoking of Ni via redissolution of surface-segregated C back into the Ni bulk. Quantitative decarburization of ZrC_*x*_@YSZ (return to O/Zr ratio of ≈1.70) via the reversal of R16 happens so fast that it cannot be resolved spectroscopically and is clearly associated with a positive current spike in Figure 2A, indicating at least partial electrochemical reversibility of ZrC_*x*_ formation at the WE giving rise to “partially reversible carbon battery” behavior (Section S13). This scenario is corroborated in Section S14 by the quantitative comparison of the current characteristics and the transported integral charges during charging and discharging (g3 vs d3 in Figure 2A). However, only the positive discharge spike can be directly used to quantify the Faradaic charge transfer during ZrC_*x*_ discharge, while the charging process in Figure S11, panels B and C of Section S14 is superimposed by a change of the ohmic background current, due to enhanced YSZ surface reduction under cathodic conditions and associated leak current (cf. Section S5).

After rapid depletion of YSZ-covering and Ni-covering carbon states, residual C_TBP_ in (d) markedly exceeds that in (a) and even exhibits a slight initial increase with time (Figure 2A, (c) → (d)). Considering the mechanism of cathodic ZrC_*x*_ growth at the WE, which is initially mediated by spillover of C_TPB_ from Ni to YSZ to form ZrC_*x*_ close to the TPB, and followed by direct CO reduction at the expanding YSZ/ZrC_*x*_ interface via R16, its full reversal should therefore involve both the reversal of R16 and “inverse” C spillover from ZrC_*x*_ toward Ni too. Figure 2B proves that this inverse C spillover can be deliberately enhanced via fast pump-down to HV, efficiently limiting NAP gas phase-mediated carbon removal during depolarization and favoring inverse spillover as the main ZrC_*x*_ depletion route.

The transition from (d) to (e) in Figure 3 reflects a combination of two effects (details in Section S15):

(a) a decrease of carbon reactivity on Ni due to R15, transforming reactive carbon species into less reactive forms and resulting in a constant Ni shielding compared to (d),
(b) the recovery of the ZrO_*y*_ signal due to reverse R9, converting accumulated carbon—located at the TPB and shielding the YSZ domains—to CO­(g), accompanied by the loss of C 1s intensity (see also Section S8).

### DFT Approaches toward the Initial Stages of C_TPB_ and C_ZrCx_ Formation in Pure CO­(g)

To better understand the thermodynamics of the observed carbide formation we computationally evaluated the energetics of hypothetical scenarios at a model from literature,^38^ that will provide trends for the presented experiments and its discussed reactions. Given the strong CO binding energy on Ni (≈1.7 eV), in addition to the energy of the strong C–O bond, the sum of both energies can be overcome only by very favorable binding sites for both carbon and oxygen at the Ni particle. Direct dissociation is, therefore, energetically unfavorable due to exceedingly high energy barriers.^39^ Instead, a disproportionation pathway is proposed, forming the even more stable molecule, CO_2_, and surface-bound carbon, which is strongly stabilized on Ni. Accordingly, carbon formation from CO­(g) was modeled in two steps: As shown in Figure 4b, an initial Boudouard-type surface reaction (R17) on Ni_7_ forms CO_2,ads_ and C_ads_ with a barrier of 1.8 eV, comparable to values for Pt.^39^

R17 $${2CO}_{ads}⇌C_{ads}+{CO}_{2,ads}$$

Only near the TPB, and only under cathodic polarization, CO_2,ads_ can be immediately reduced to CO_ads_ on Ni sites and ionic O^2–^ species filling a nearby YSZ vacancy (R18), whereby a desorption and readsorption of CO_2_ at the vacancy-rich TPB appears possible.

R18 $${CO}_{2,ads}+{2e}^{-}⇌{CO}_{ads}+O^{2-}$$

Here, CO_2,ads_ serves as a reactive intermediate for R18, while the remaining C_ads_ is observed as C_TPB_ experimentally. Combining reactions R9 (cf. R17) and R2 (cf. R18) results in R3 (CO + 2e^–^ ⇌ C + O^2–^), while CO_2,ads_ acts as a barrier-decreasing intermediate.

**Figure 4:**
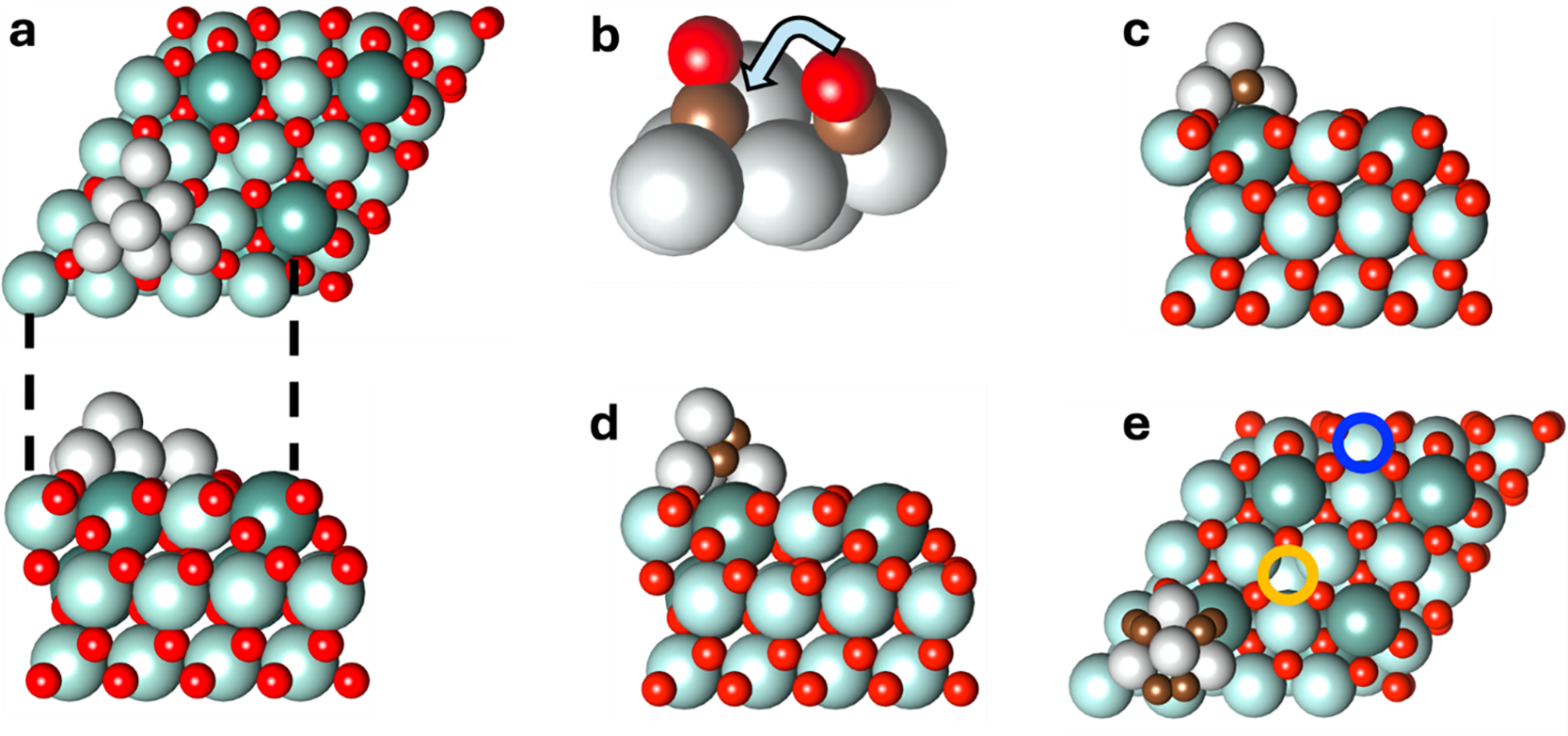
Computational models for initial stages of C_TPB_ and C_ZrCx_ formation in pure CO­(g). (a) Top- and side view of the Ni_7_@YSZ model system and (b) CO disproportionation step forming CO_2,ads_ and C_ads_ at the Ni_7_ entity, (c) one carbon atom at a Ni_4_ particle, (d) two carbon atoms at Ni_4_, and (e) six carbon atoms at Ni_4_ and possible C adsorption sites on YSZ (orange—initial adsorption site, blue—secondary adsorption site). Ni—light gray, Zr—cyan, Y—petrol, C—brown, O—red.

To understand the energetics of ZrC_*x*_ formation, the carbon adsorption energy on YSZ was modeled as a function of carbon saturation of a Ni_4_ cluster (Figure 4c–e). Considering an accumulation of vacancies (beyond Y^3+^ doping), C transfer from Ni_4_ to YSZ is solely ≈1 eV uphill—experimentally surmountable with ≈0.9 V polarization (Section S16). Since vacancy accumulation near the TPB and carbon supersaturation are experimentally accessible, negligible reaction barriers are expected for subsequent Zr carburization.

Summarizing the transfer of carbon from the Ni particle to the YSZ needs two preconditions: local accumulation of vacancies at the YSZ surface near the TPB and sufficiently carbon-saturated Ni. As such the accumulation of vacancies near the TPB on YSZ is clearly field-driven and viable due to sufficient anion mobility at 600 °C and applied polarization.^43^ However, an already surface reduced YSZ supports the formation of an extended ZrC_*x*_ layer (cf. Section S12: The degree of ZrC_*x*_ formation correlates with the CO partial pressure.). Finally, the initial accumulation of vacancies is an electrochemical process, the carbon spillover is a thermochemical step, and the reduction of formally Zr^4+^ species is purely electrochemical. The growth of ZrC_*x*_ after this initial formation is an electrochemical reaction according to R16 (cf. Section S14).

### Reactivity of C_TPB_ with H_2_

As methane formation according to reaction R7 (C + 2H_2_ ⇌ CH_4_) has been discussed in the literature,^2,11^ reactivity considerations of C_TPB_ toward H_2_ are evident. As shown in Figure 5, hydrogen coadsorption is not accompanied by accelerated C hydrogenation to CH_4_—despite efficient hydrogen adsorption kinetics on metallic Ni^44^—but instead impedes C_TPB_ dissolution. To probe the respective carbon dissolution kinetics, Section S17 compares the decrease in the C_TPB_/Zr ratio under *H*_2_(*g*) with HV conditions.

**Figure 5:**
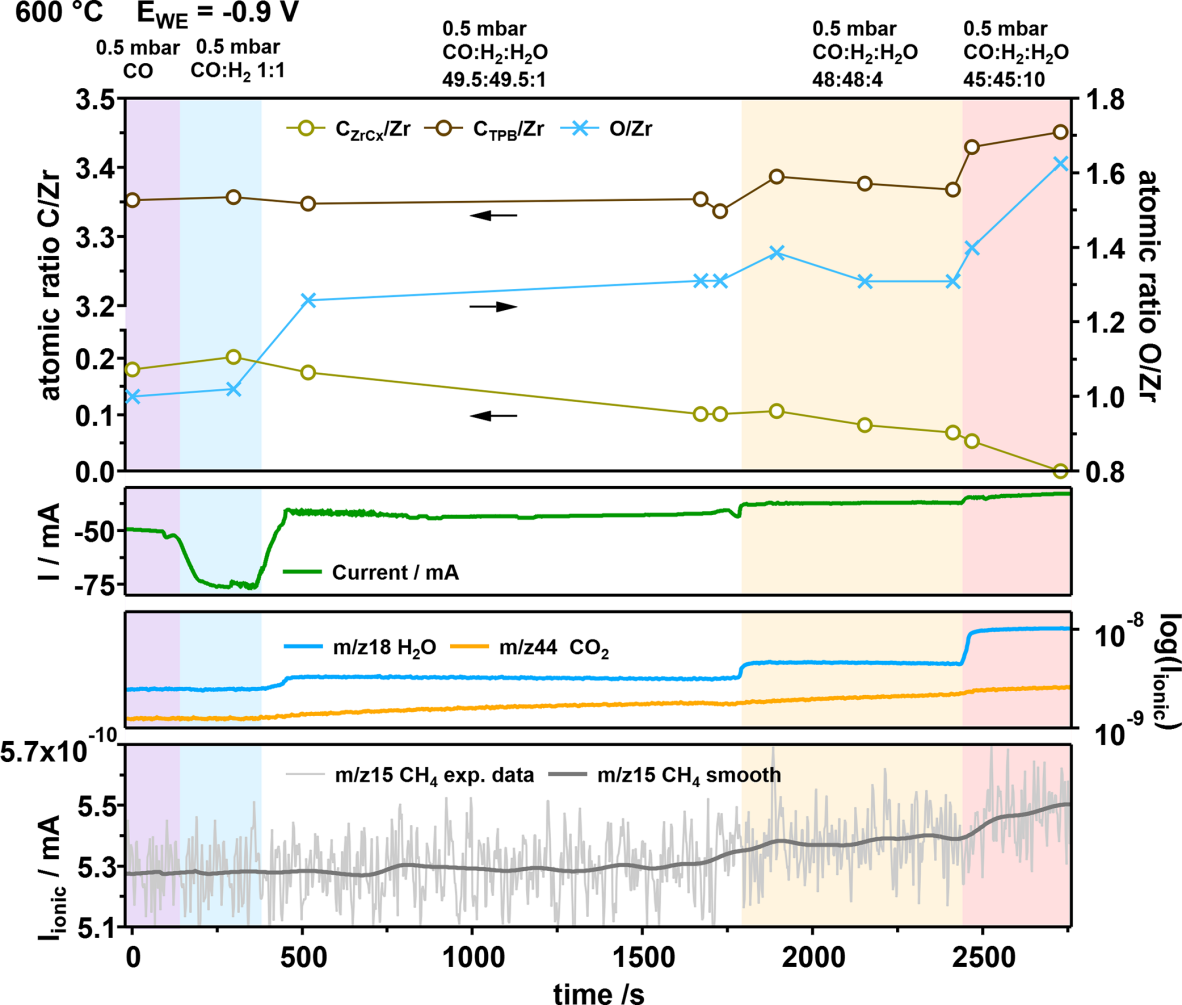
Hydrolysis of polarization-induced C_ZrCx_ to methane is monitored in a CO/H_2_/H_2_O atmosphere. Conditions simulate strongly reducing co-electrolysis (high cathodic potentials and current densities), where local CO and H_2_ partial pressures rise near the TPB via enhanced forward reactions R2 and R4. After ZrC_*x*_ formation and CO/H_2_ establishment, small amounts of H_2_O were added. Shaded regions indicate gas compositions: pure CO (violet), CO/H_2_ = 1:1 (blue), and CO/H_2_ = 1:1 with increasing H_2_O partial pressures −5 × 10^–3^ (white), 2 × 10^–2^ (yellow), and 5 × 10^–2^ mbar (red). The surface response was tracked via atomic ratios C_ZrCx_/Zr (light green), C_TPB_/Zr (brown), and O/Zr (light blue), correlated with electrochemical currents and mass spectrometry, at constant *T* = 600 °C and *E*_WE_ = −0.9 V. Mid and bottom panels shows *E*/*I* characteristics (dark green) and MS signals of H_2_O (*m*/*z* = 18; blue), CO_2_ (*m*/*z* = 44; orange), and CH_4_ (*m*/*z* = 15, gray).

### Reactivity of Polarization-Induced C_ZrCx_ with H_2_/H_2_O

To reveal a potential mechanistic role of the polarization-stabilized C_ZrCx_ species with respect to electrochemical methane formation, we tested the activity of ZrC_*x*_ toward both H_2_ and H_2_O vapor, while simultaneously tracking for CH_4_ by online mass spectrometry. In this context, *m*/*z* = 15 (CH_3_^+^) is the only fragment which is expected to increase due to CH_4_ formation as it stems exclusively from methane. The *m*/*z* = 16 signal is unspecific, as it is superimposed by O^+^ fragments from the dominant CO background and is also influenced by H_2_O and CO_2_ pressure changes over the course of the experiment.

The scenario of strongly reducing co-electrolysis conditions exhibiting high CO/H_2_ levels was simulated using the experimental design shown in Figure 5. After the ZrC_*x*_ species was created in CO­(g), the gas composition was changed to a 1:1 mixture of CO and H_2_ in order to identify a potential reactivity of ZrC_*x*_ toward H_2_. At this point, neither a reactive depletion of the ZrC_*x*_ state nor methane gas formation were detected, indicating that the carbide is largely unreactive toward hydrogen. The inert behavior of C_ZrCx_ against CO/H_2_ mixtures and pure H_2_ was confirmed by extensive investigations outlined in Section S18. In due course, a small amount of H_2_O vapor was added while monitoring the surface composition (atomic ratios of C_ZrCx_/Zr, C_TPB_/Zr, and O/Zr) by XPS next to the formation of CH_4_ (R11) and CO_2_ (R13) by QMS and the current characteristics. The water vapor content was then increased in a stepwise manner to hydrolyze the carbide.

The current increase observed upon addition of *H*_2_(*g*) to pure CO­(g) (≈160 s) is most likely due to the enhanced YSZ surface reduction and the associated leak current (Section S5). However, as soon as minute amounts of H_2_O are added, the leak current is strongly diminished, due to the reoxidation of the YSZ surface by water, and approaches a stable level after several minutes. Upon the first addition of H_2_O (5 × 10^–3^ mbar), the ratio of C_TPB_/Zr remains constant, and only a moderate increase of the *m*/*z* = 15 and 44 signals are detected. The O/Zr ratio increases, whereas the C_ZrCx_/Zr ratio decreases, indicating the partial hydrolysis of the ZrC_*x*_ surface species by water. Upon increasing the H_2_O vapor pressure stepwise, CH_4_ (R11) and CO_2_ (R13) start to form, while the consumption of the carbide becomes quantitative and was accompanied by a substantial reoxidation of the YSZ surface. The observed slow increase in the C_TPB_/Zr ratio is most likely related to the ongoing deposition of C_TPB_ via R3.

Uchida et al.^20^ measured a C1 product distribution in the ZrC hydrolysis reaction at 700 °C, resulting in a CO_2_/CH_4_ ratio of 100:1.7. Approximately the same ratio was found by assessing the relative increase of the *m*/*z* = 15 signal to the *m*/*z* = 44 signal.

## Conclusion

Based on the observed interconversion of C_TPB_ and C_ZrCx_ on Ni metal and 8-YSZ and the observed electrocatalytic properties, we conclude that CH_4_ formation is enhanced by hydrolysis of a ZrC_*x*_ species on YSZ, rather than by direct hydrogenation of C_ZrCx_ or C_TPB_. This applies specifically under strongly cathodic CO_2_/H_2_O co-electrolysis conditions with a significant fraction of CO and H_2_. The role of the dynamic redox surface chemistry of the YSZ solid electrolyte is often overlooked in the metal-focused discussion of methanation activity and, more generally, of the electrocatalytic electrode performance toward CH_4_, representing an entirely unexpected mechanistic aspect for hydrocarbon-forming electrode pathways.

The proposed mechanism in Figure 6 summarizes the findings of sequential Ni carburization (a–d), ZrC_*x*_ growth (e), and enhanced methane formation via ZrC_*x*_ hydrolysis in the presence of H_2_O­(g) (f).

**Figure 6:**
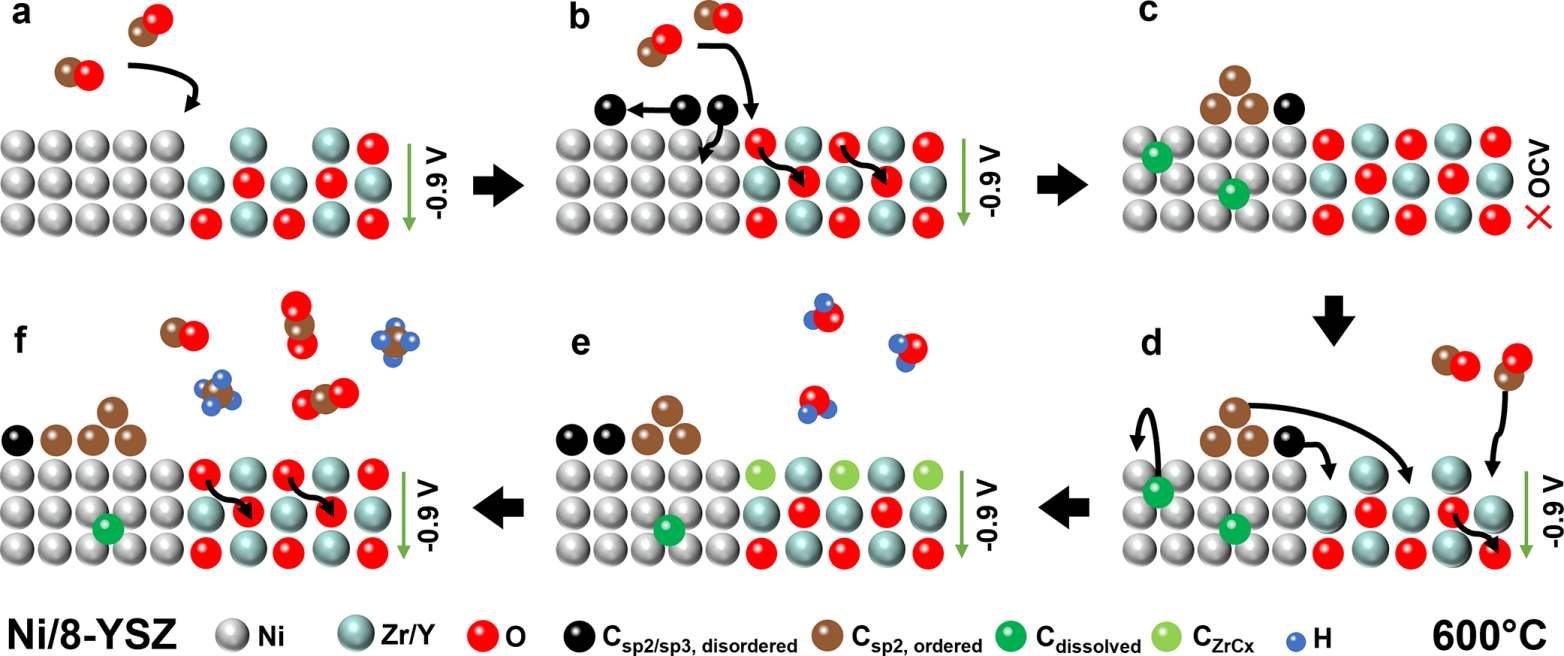
Based on the experimental and theoretical results a reaction mechanism is proposed for the direct methane generation pathway in high-temperature CO_2_/H_2_O co-electrolysis on Ni/YSZ interfaces. (a) Cathodic polarization creates an oxygen-deficient YSZ state at the TPB in CO­(g). (b) CO­(ads) electrolysis forms C_sp_2_/sp_3_/disordered_ via Boudouard-type CO activation and oxygen vacancy quenching (sequence R17 and R18 yields R3, DFT results). (c) Upon depolarization, C_sp_2_/sp_3_/disordered_ ripens to C_sp_2_/ordered_ (R15), while Ni becomes saturated with C_dissolved_. (d) Repolarization drives field-induced C_dissolved_ exsolution from Ni and C_TPB_ spillover to YSZ, forming ZrC_*x*_ at reduced/defective YSZ. Concurrently, CO induces direct YSZ carburization (R16). (e) ZrC_*x*_ reacts with H_2_O­(g), forming ZrO_*y*_ via hydrolysis. (f) Hydrolysis yields CO, CO_2_, and CH_4_, while unreactive C_sp_2_/ordered_ ripens (R15).

Li et al.^11^ investigated CO_2_/H_2_O co-electrolysis and proposed a high-voltage reaction pathway based on R7 to explain the detected CH_4_ formation, also discussed in a recent review.^2^ Their established distribution of C-products led to a CO_2_/CH_4_ ratio of 100:0.6 at 650 °C, which was remarkably close to that determined in this study (100:1.7). The illustrated hydrolytic carbide-mediated mechanism toward CH_4_ represents an unexpected alternative route, which is spectroscopically inaccessible on technological 3D-porous cermet electrodes. Only the combination of a tailor-made 2D-porous thin-film electrode approach^23^ with operando NAP-XPS^24^ allows to uncover such unprecedented mechanistic insights. Advancing the field requires a focus on spectroscopy-optimized electrode development with abundant electrolyte-metal phase boundaries, enabling dynamic carbon exchange and high availability of reactive carbon species for hydrocarbon formation. In particular, beneficial carbon spillover properties and electrolytes with CH_4_-selective carbide hydrolysis chemistry should be explored.

## Supplementary Material



## Data Availability

The data is publicly accessible under the DOI: 10.6084/m9.figshare.30174544.

## Notes

Reaction network and WE characterization and setup; electrochemical formation and accumulation of C_TPB_; carbon growth on Ni domains and Zr 3d shielding; I/E curves; current characteristics under chronoamperometric conditions (E_WE_ = −0.9 V, constant); imaging carbon growth and dissolution; carbon dissolution kinetics in the Ni electrode film; C_TPB_ as a prerequisite for ZrC_*x*_ formation; detailed C 1s analysis; presence of “oxycarbidic” zirconium and Zr 3d fit model, validation of partially reversible spillover of C_TPB_ to YSZ; gas phase-dependent comparison of ZrC_*x*_ formation; partially reversible carbon battery; detailed current characteristics of ZrC_*x*_ formation and decay; detailed interpretation of final trends in Figure 3; energetics of carbon adsorption at Ni_4_ and ZrO_*x*_; dissolution rate of C_TPB_ and reactivity of C_TPB_ with H_2_; and reactivity of polarization-induced C_ZrCx_ with H_2_ (PDF)
