# Supplementary material for: Unexpected Reactivity of Zirconium Carbide on Nickel/Yttria-Stabilized Zirconia Boosts CO2/H2O Co-electrolysis to Methane
Source: Chem Mater. 2026 Apr 30;38(15):7637–48. doi: 10.1021/acs.chemmater.6c00480 (PMC13471733; doi:10.1021/acs.chemmater.6c00480)
Supplement: Supplementary file 1 [file cm6c00480_si_001.pdf]

Supporting Information

Unexpected reactivity of zirconium carbide on nickel/yttria-stabilized zirconia boosts CO_2/H_2O co-electrolysis to methane

Christoph W. Thurner[1,4], Kevin Ploner[2], Patrick Obendorf[1], Daniel Winkler[1], Alexander Genest[3,*], Günther Rupprechter[3], Simon Penner[1] and Bernhard Klötzer[1,*]

[1]*Institute of Physical Chemistry, University of Innsbruck, Innrain 52c, A-6020 Innsbruck, Austria*

[2] *Plansee SE, Metallwerk-Plansee-Straße 71, 6600 Reutte, Austria*

[3]*Institute of Materials Chemistry, Technische Universität Wien, Getreidemarkt 9/BC/01, Vienna 1060, Austria*

[4]*Ceratizit S.A., Rue de Holzem 101, 8232 Mamer, Luxembourg*

[*] Corresponding Authors: [E-mail: Bernhard.Kloetzer@uibk.ac.at, Alexander.Genest@tuwien.ac.at]

Contents

S1: WE characterization and setup 3

S2: Electrochemical formation and accumulation of C_{TPB} 4

S3: Carbon growth on Ni domains and Zr 3d shielding 6

S4: I/E curves 7

S5: Current characteristics under chronoamperometric conditions (E_{WE} = -0.9V, constant) 8

S6: Imaging carbon growth and dissolution 9

S7: Carbon dissolution kinetics in the Ni electrode film 10

S8: C_{TPB} as prerequisite for ZrCx formation 12

S9: Detailed C1s analysis 13

S10: Presence of "oxycarbidic" zirconium and Zr 3d fit model 14

S11: Validation of partially reversible spillover of C_{TPB} to YSZ 15

S12: Gas phase-dependent comparison of ZrC_x formation 16

S13: Partially reversible carbon battery 17

S14: Detailed current characteristics of ZrC_x formation and decay 18

S15: Detailed interpretation of final trends in Figure 3 19

S16: Energetics of carbon adsorption at Ni_4 and ZrO_2 21

S17: Dissolution rate of C_{TPB} and reactivity of C_{TPB} with H_2 24

S18: Reactivity of polarization-induced C_{ZrCx} with H_2 27

References 30

S1: WE characterization and setup

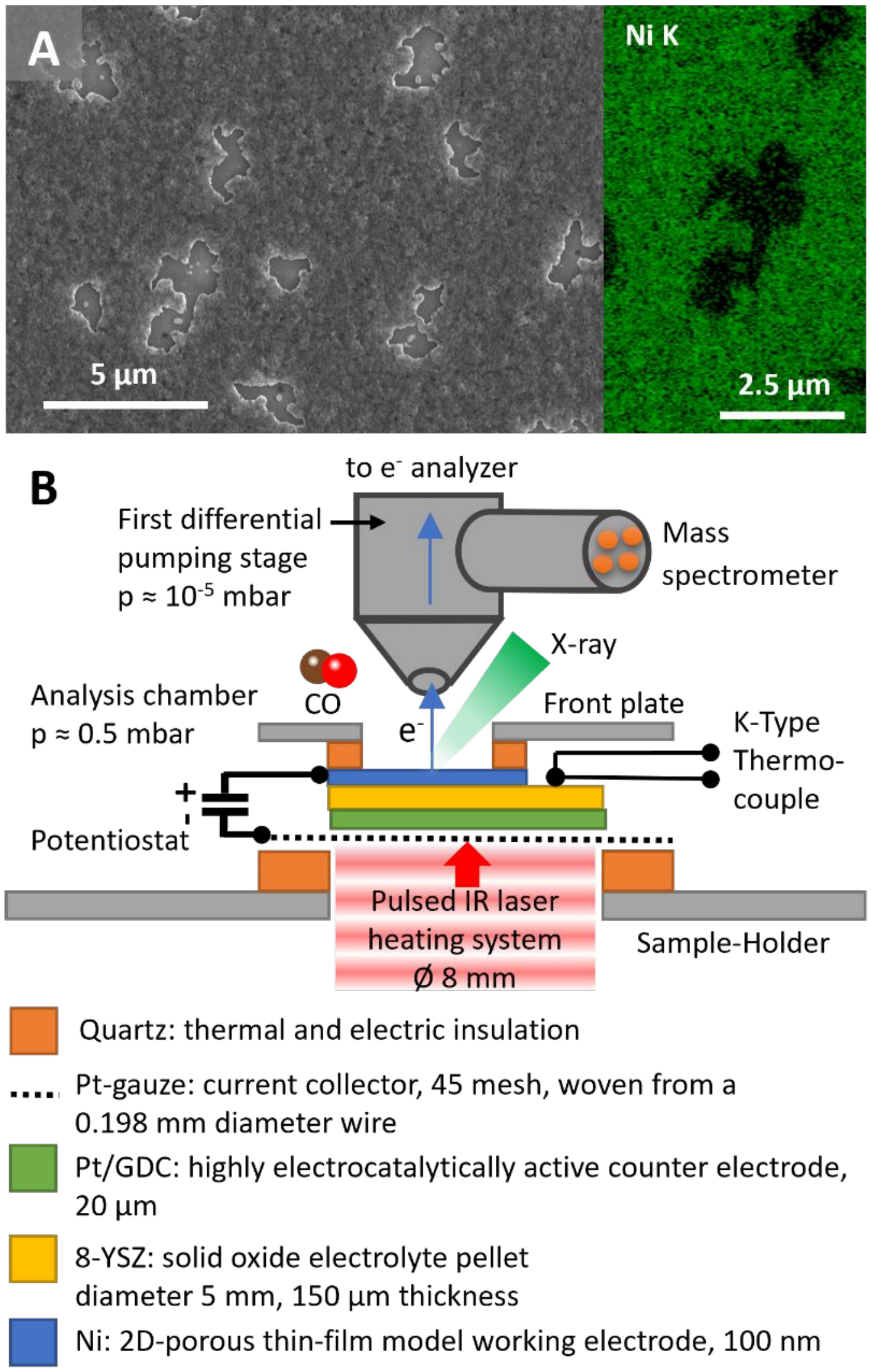

Figure S1. A: SEM image and EDXS map of the annealed 2D-network-like Ni thin-film showing a local de-wetting of the YSZ electrolyte. B: Schematic cross-section of the solid oxide electrochemical cell mounted on the sample holder in the NAP-XPS chamber (p = 0.5 mbar CO). X-ray excited photo electrons are leaving the sample, enter the differentially pumped column via a nozzle and are guided by lens systems toward the energy analyzer. The first differential pumping stage exhibits an operating pressure in the 10^{-5} mbar range suitable for a mass spectrometry, detecting gas species generated on top of the sample without any delay. The heating system is based on an infra-red laser, which power is controlled by a K-type temperature sensor attached to the sample surface and allows precise sample heating.

S2: Electrochemical formation and accumulation of C_{TPB}

Carbon growth via reaction R3 was electrochemically induced by applying a cathodic potential to the sample, which was kept isothermally at 600 °C in a 0.5 mbar CO atmosphere. The applied cathodic potential (E_{WE}) was increased step-wise from 0 V to -0.9 V (step size 0.1 V), while tracking the C 1s region. At each voltage step, a set of eleven C 1s spectra was recorded over a period of ≈10 min. After staying at -0.9 V for 312 s, a C_{TPB} component assigned to C_{sp3} appears at a BE of 285.0 eV[1] (Figure S2A). During further growth, the C_{TPB} peak increases in each spectrum and shifts toward a graphite-like form of carbon (C_{sp2}) at a BE of 284.6 eV. In total, 23 C 1s spectra were recorded within ≈1200 s under constant polarization conditions, while the transported electrical charge Q was monitored as a function of time (Figure S2A, inset). A control experiment illustrated in Figure S2B is proving the absence of carbon deposition in CO(g) under unpolarized conditions over time (≈100 min).

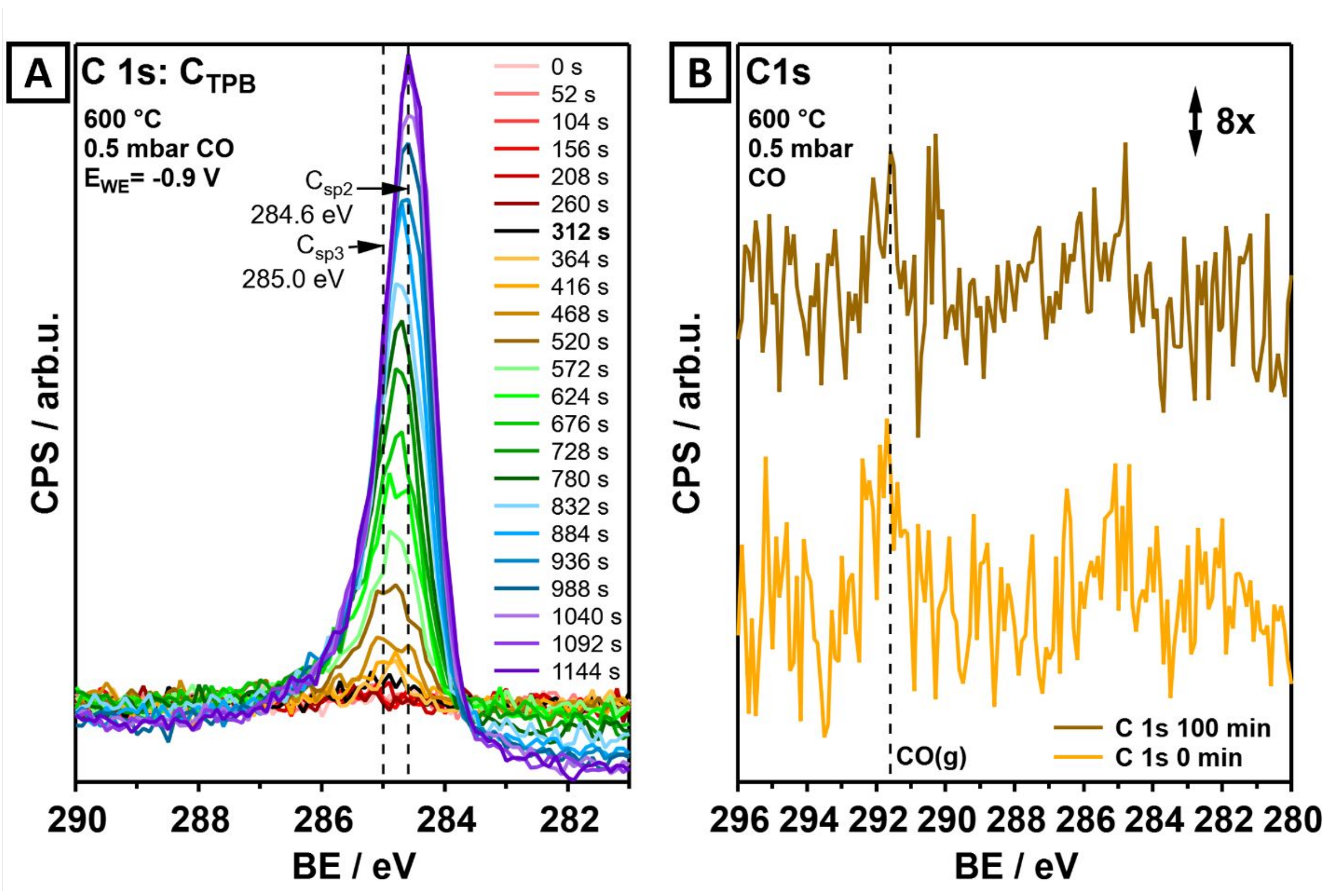

Figure S2. Panel A: C 1s spectra recorded at 600 °C in 0.5 mbar CO at E_{WE} = -0.9 V and plotted as a function of time. The inset shows the corresponding integral transferred charge. C_{TPB} appears initially as C_{sp3} at a BE of 285.0 eV and is then shifted toward C_{sp2} (BE = 284.6 eV) during carbon growth, as indicated by the dashed

lines. Panel B: Control experiment showing the absence of carbon deposition in CO(g) in the unpolarized state over time (≈100 min).

S3: Carbon growth on Ni domains and Zr 3d shielding

The comparison of the Ni 2p, Zr 3d and C_{TPB} integral peak areas during the first cycle (g1) of carbon growth on identically prepared sample S1 and S2 in Figure S3 results in a rather constant Zr 3d intensity, while the increasing amount of carbon is associated with a simultaneous decrease of the Ni 2p intensity. The slight decrease of the integral peak area of Zr 3d at high carbon coverages is assigned to a geometrical photoelectron shielding effect of the Ni-covering carbon with increasing layer thickness, which seems to be more pronounced on sample S2 (panel B). Although conditions are the same, the carbon growth process on S1 is much faster (≈550 s) as compared to S2 (≈1300 s). The time resolution in panel B is lower than in A due to the additional recording time for the Ni 2p signal.

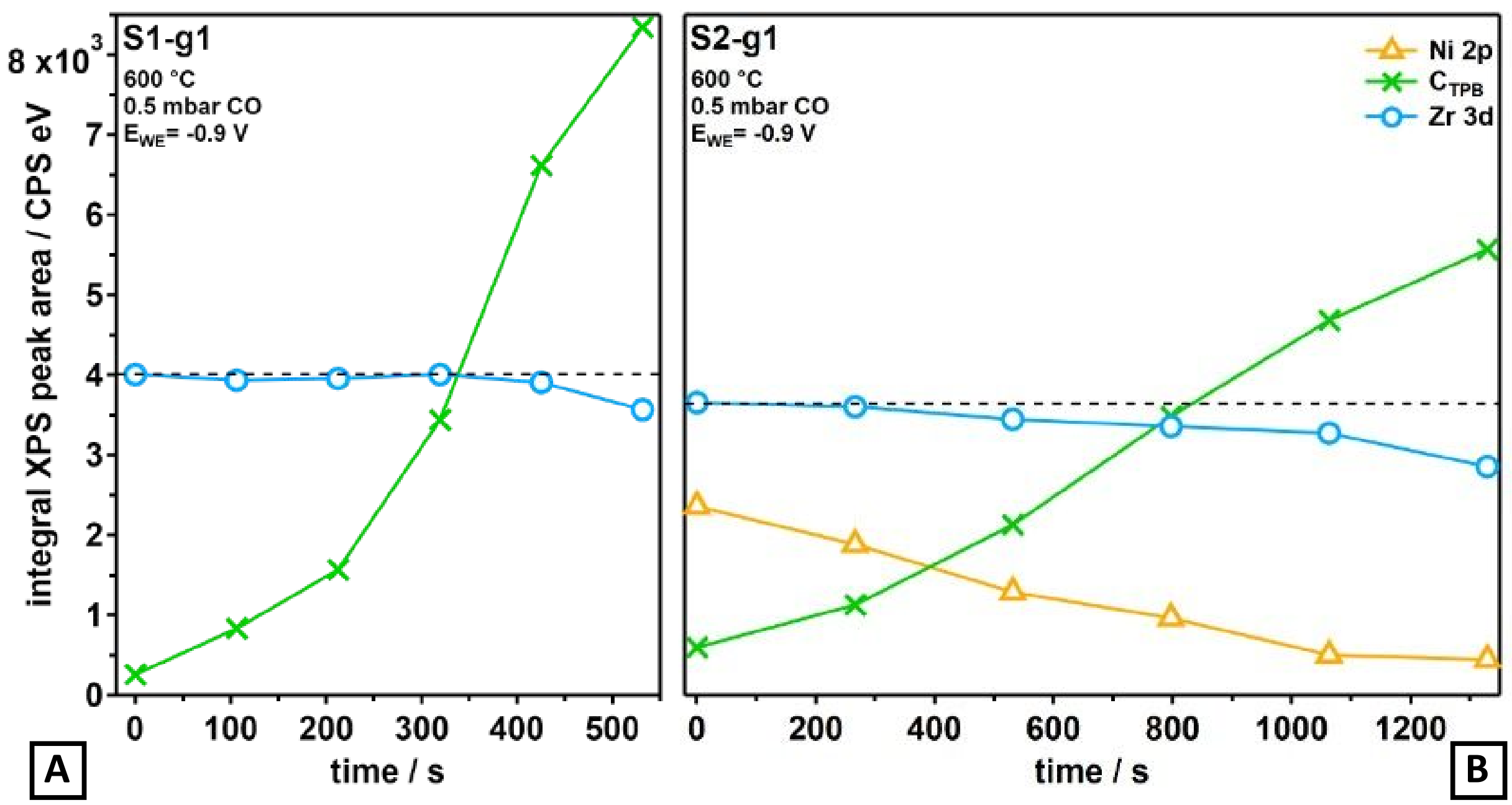

Figure S3. A/B: The integral peak areas of C_{TPB} (green), Zr 3d (blue) and Ni 2p (orange) are tracked as a function of time during the first cycle (g1) of electrochemically induced growth of carbon on two samples (S1 and S2).

S4: I/E curves

The experiments shown in Figure S4 support the initially predominant carbon growth on the Ni domains. The gradually decreasing cathodic currents in a cycling potential sweep experiment is a consequence of a progressive blocking of C_{TPB}-forming boundary sites.

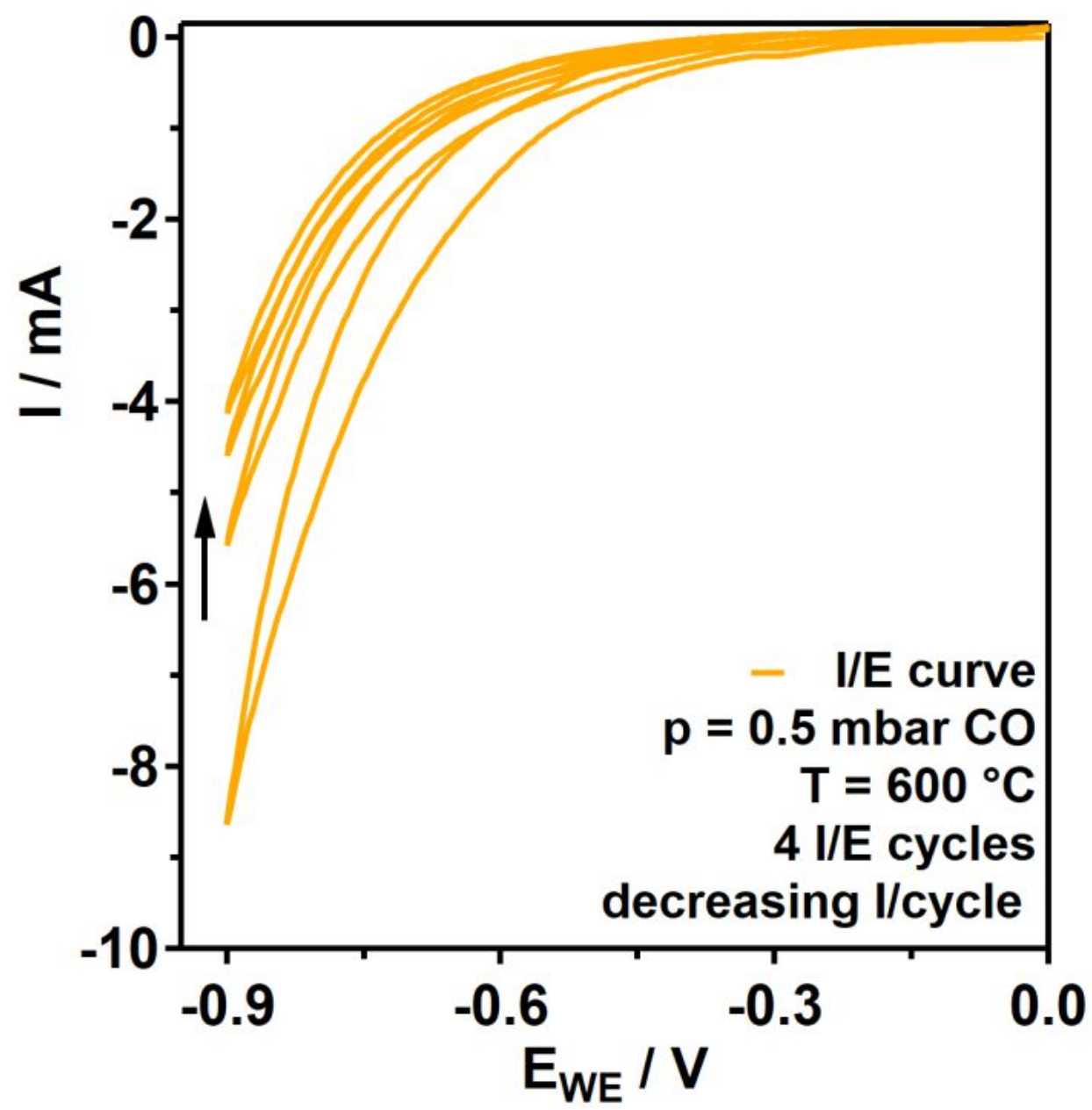

Figure S4. Linear sweep I/E curves (cyclic voltammograms) recorded in 0.5 mbar CO at 600 °C with a scan speed of 20 mV/s. The first I/E curve shows a maximum current of ≈-8.5 mA measured at -0.9 V. The maximum current decreases with each I/E cycle, approaching ≈-4.0 mA after four cycles and thereby suggesting an increase in accumulated TPB site-blocking carbon species, limiting the current.

S5: Current characteristics under chronoamperometric conditions (E_{WE} = -0.9V, constant)

Upon maintaining a constant voltage of -0.9 V during a chronoamperometric experiment, the cathodic current first drops due to local C_{TPB} accumulation, but slowly increases over time in due course (Figure S5). This is most likely caused by enhanced local surface reduction of the electrolyte and formation of electron-conducting YSZ surface states e.g. at the circumference, i.e. the uncovered side walls of the disc-shaped YSZ pellet substrate. This gives rise to an electronic leak current superimposed on the faradaic electrochemical processes at the electrodes. By turning the voltage on, the current exhibits fast initial damping (C accumulation via R3), followed by a slow increase, which is attributed to progressive formation of reduced Zr surface species, acting as electron donors for enhanced ohmic surface/interface conductivity.

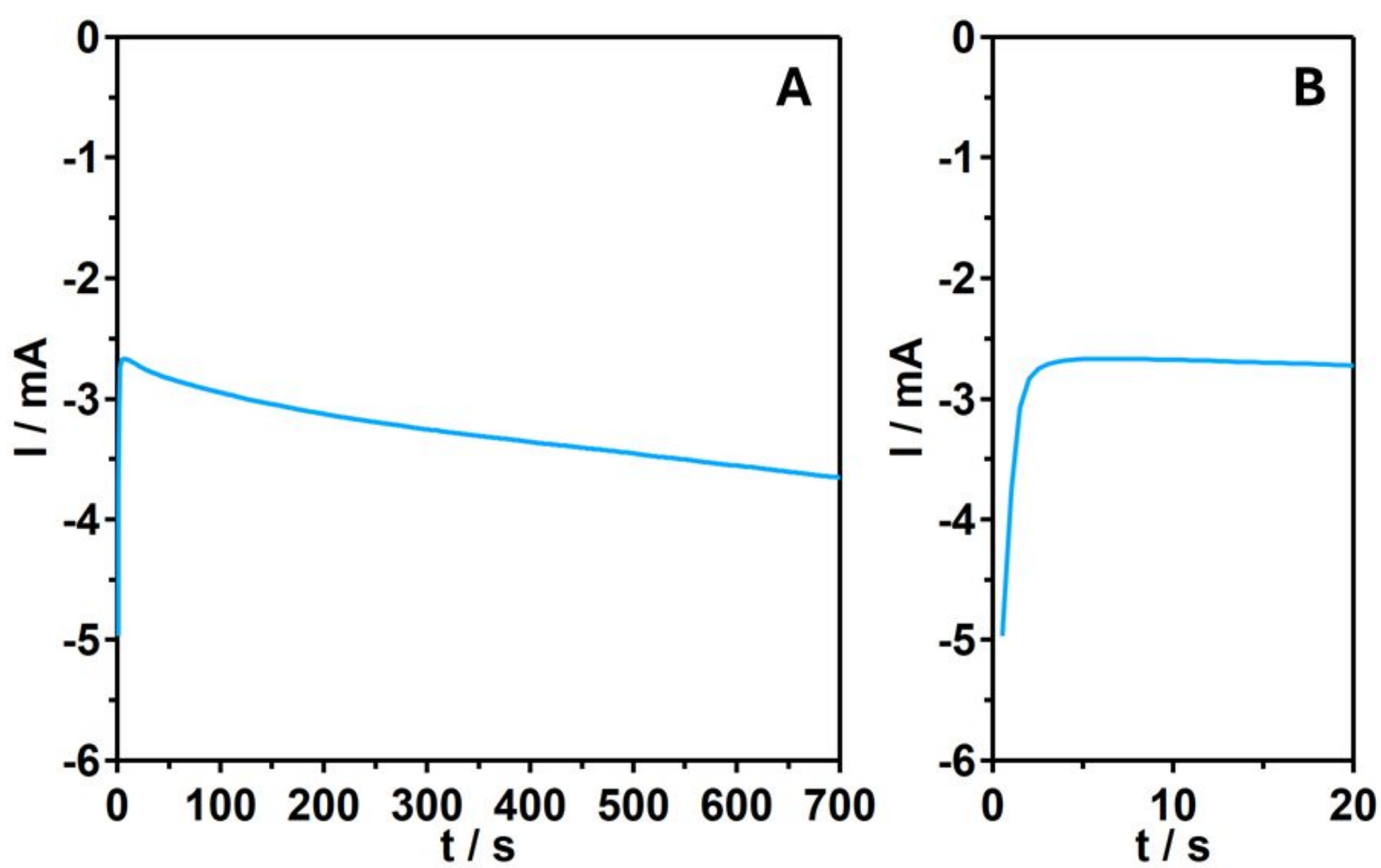

Figure S5. Current response upon constant cathodic polarization at E_{WE} = -0.9 V recorded in 0.5 mbar CO at 600 °C.

S6: Imaging carbon growth and dissolution

During cathodic polarization, the first cycle (g1) of carbon growth on sample S1 was also clearly visible as a progressing, sample-darkening front in the control camera of the instrument (Figure S6). The time scale of 890 s for growth is higher than that presented in Figure S3A because the growth started quite locally and caused no C 1s intensity increase, at least with respect to the first ≈300 s. After switching back to the unpolarized state, the process appears reversible, indicating relatively fast re-dissolution and/or clean-off of the C-deposits within ≈550 s.

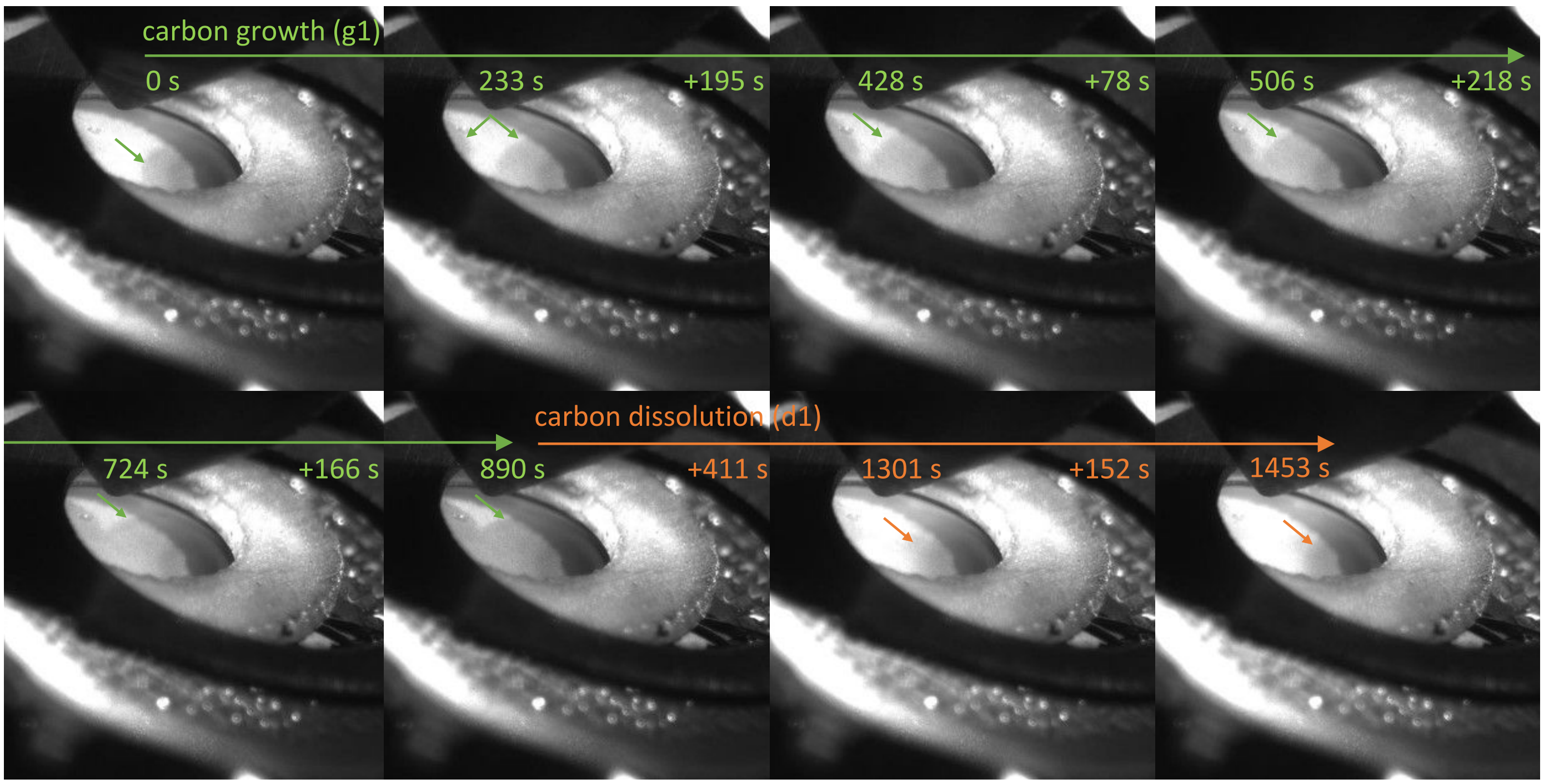

Figure S6. Series of images taken with the NAP-XPS sample control camera on sample S1, illustrating the growth front of the first cycle of electrochemically deposited carbon (g1) at E_{WE} = -0.9 V and its vanishing at open circuit voltage (OCV).

S7: Carbon dissolution kinetics in the Ni electrode film

After growing C_{TPB} on each sample (S1 and S2) for ≈1300 s, while recording the C 1s intensity, the potential was switched to OCV and the NAP-XPS chamber was immediately pumped down to $5 \cdot 10^{-8}$ mbar to avoid carbon clean-off reactions while studying carbon dissolution in Ni. Upon reaching this pressure (≈180 s), the next C 1s spectrum, followed by a Zr 3d spectrum, was recorded. On pristine sample and during the first carbon dissolution cycle (S2-d1), the C_{TPB}/Zr ratio drops quickly during and reaches values around 0.2 already after 500 s, indicating relatively fast C dissolution and a still high C storage capacity of the Ni layer (Figure S7). The decrease on the C-pre-saturated sample during the third dissolution cycle (S1-d3) is much slower and stagnates at a C_{TPB}/Zr ratio of 2.2 after 800 s and 2.1 after 1200 s, due to an almost "full" $C_{dissolved}$ reservoir.

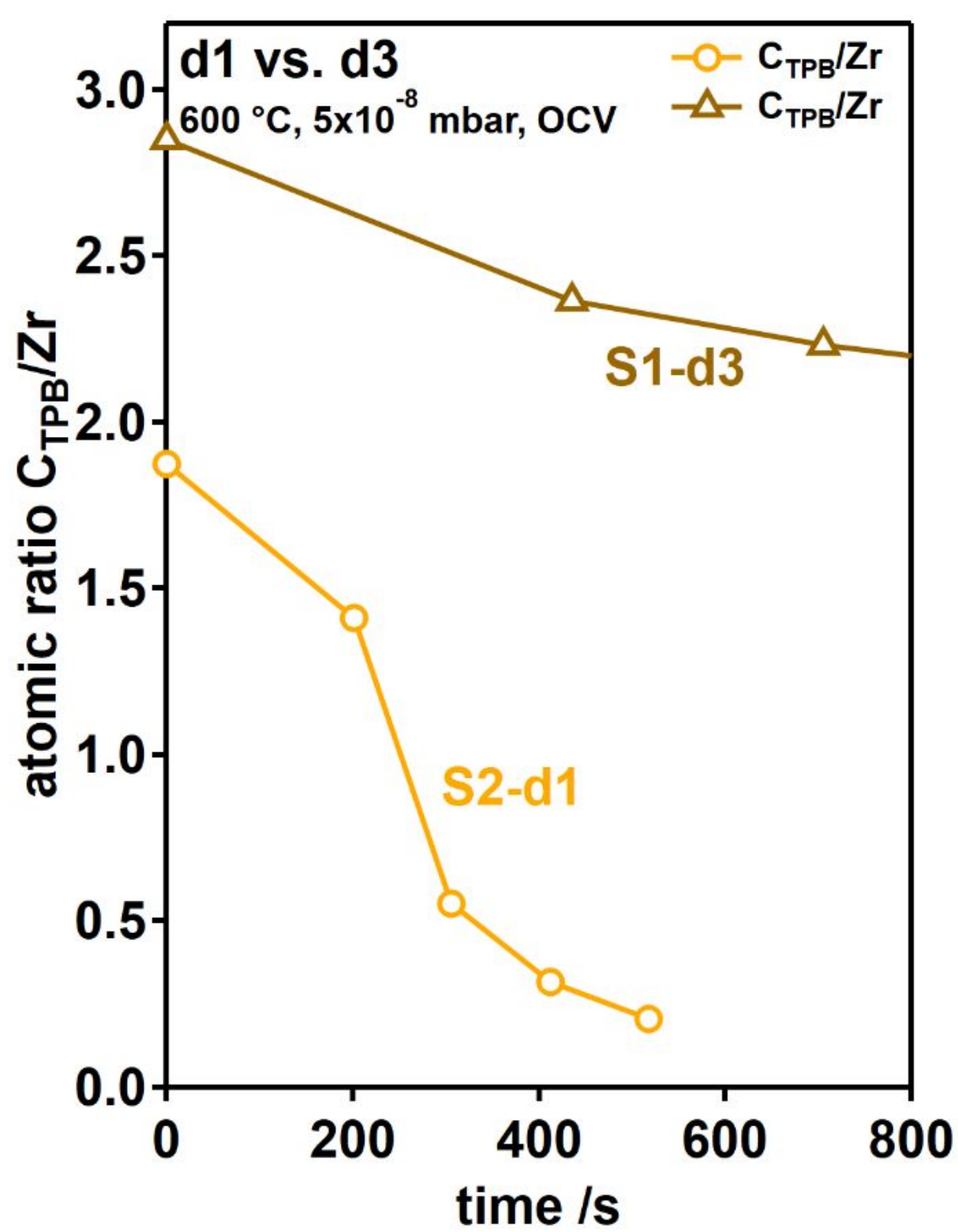

Figure S7. Bulk diffusion kinetics of carbon in the Ni electrode film derived from the atomic ratio C_{TPB}/Zr as a function of time. C 1s intensity loss on a pristine sample (S2-d1) and an already carbon pre-saturated sample (S1-d3), which underwent three preceding cycles of carbon growth (g3) and dissolution (d3). After growing C_{TPB} for ≈1300 s, while recording the C 1s intensity, the potential was switched to OCV and the

NAP-XPS chamber was immediately pumped down to $5 \cdot 10^{-8}$ mbar (≈180 s), in order to avoid clean-off reactions while studying carbon dissolution in Ni. Then, a series of C 1s spectra, followed by the respective Zr 3d spectra (for intensity normalization) was recorded.

S8: C_{TPB} as prerequisite for ZrCx formation

The rather fast decrease of the C_{TPB}/Zr ratio of d2, which occurs upon depolarization in the CO gas phase, is expected to be a combination of rather slow C dissolution anticipated on a carbon pre-saturated Ni film and reverse reaction R9 ($2CO \rightleftharpoons C + CO_2$). R9 is concluded, because online QMS under cathodic polarization of the WE proved the preceding accumulation of CO_2 in the analysis chamber with time, as each C atom generated via R3 at the WE must produce one CO_2(g) molecule via reverse reaction R2 at the CE. The datapoints of trace d1, d2, and d3 at ≈500 s reveal an increasing coverage of the Ni domains by C_{TPB} after each cycle. Following trace S2-d3, a steep decrease of C_{TPB}/Zr at the beginning, continued by a rather constant coverage of C_{TPB}, suggests the presence of two forms of carbon. One reactive form is suspected to originate from freshly grown disordered carbon and a rather unreactive form, most likely a graphite-like reaction product, from R15. The indices (a), (d) and (e) represent the same data as presented in Figures 2, 3, S8 and S9.

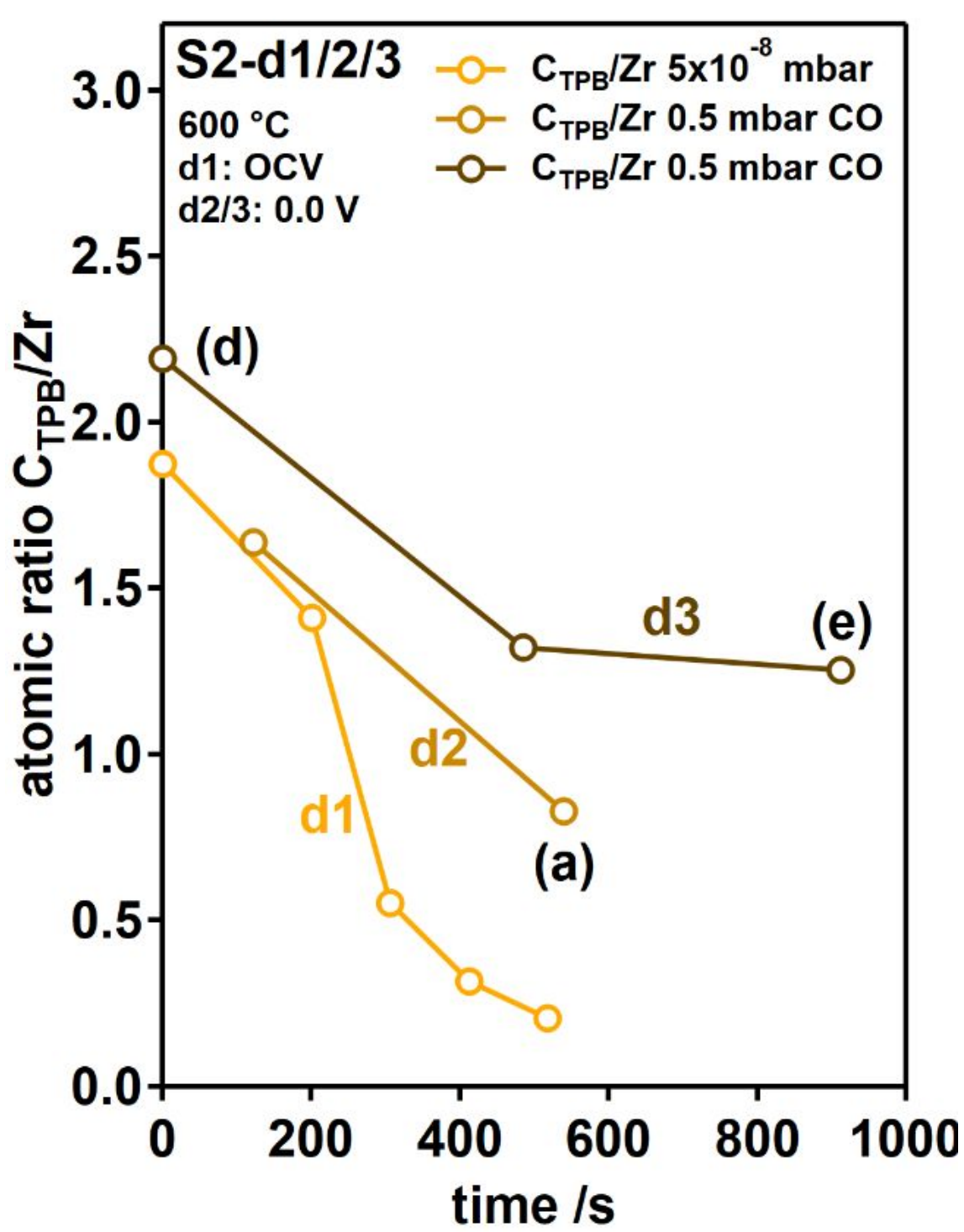

Figure S8. The atomic ratio C_{TPB}/Zr illustrating the degree of C_{TPB} coverage of sample S2 is plotted over time, comparing three carbon dissolution or decomposition cycles.

S9: Detailed C1s analysis

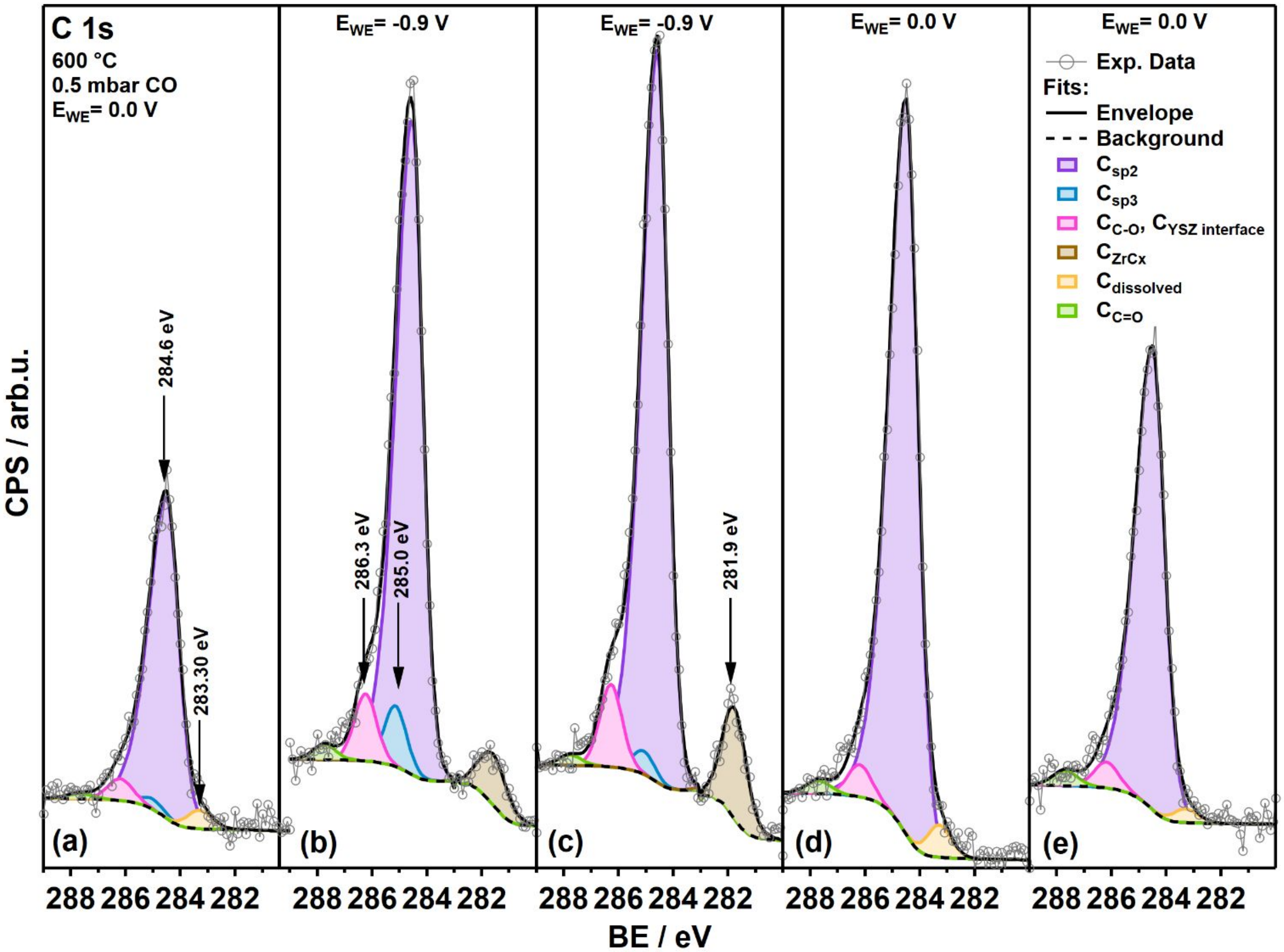

Figure S9. XP spectra series of the C 1s region recorded under OCV conditions and -0.9 V cathodic polarization at 600 °C in 0.5 mbar CO, showing a magnification of the data presented in Figure 3. The peak analysis, based on a fitting routine established by Biesinger[1], is focused on the time development of the components C_{sp2} (violet), $C_{C\text{-}O}/C_{YSZ\ interface}$ (pink), C_{ZrCx} (brown), C_{sp3} (blue), $C_{dissolved}$ (orange) and $C_{C=O}$ (green).For C_{sp2} component an asymmetric peak shape was used, while the other components were fitted with a symmetric GL(30) peak.

S10: Presence of "oxycarbidic" zirconium and Zr 3d fit model

The $C_{YSZ,interface}$ component detected in Figure 3 and S9 suggests the presence of an Zr oxycarbide interdiffusion layer with gradually variable ZrC_xO_y coordination. By restricting the full-width-half-maximum (FWHM) of the ZrO_y peaks to a maximum value of 1.64 eV, - extracted from the fit of purely oxidic Zr 3d states detected under non-carbide conditions (e.g., Figure 3 (a), (d) and (e)) - intermediate BE components between the purely carbidic and oxidic Zr 3d components were required to fit the Zr 3d spectra. Based on the fitting model established in Ref. 2, asymmetry was assigned to the ZrC_x line shape (i.e., GL(30)T(1.3) line shape cf. Ref. 2), while the FWHM was capped with 1.20 eV. The FWHM of the ZrC_xO_y component was restricted to a maximum of 1.80 eV. For all components the area ratio of the spin-orbit coupling ($d_{3/2}$ to $d_{5/2}$) was set to 2:3 and spin-orbit splitting was fixed with 2.4 eV. The established results agree with a recent study on thin ZrC_xO_y films, which reports that bulk-like Zr "oxycarbide" species are associated with the simultaneous presence of spectroscopically distinct oxidic, sub-oxidic and carbidic Zr components[2].

S11: Validation of partially reversible spillover of C_{TPB} to YSZ

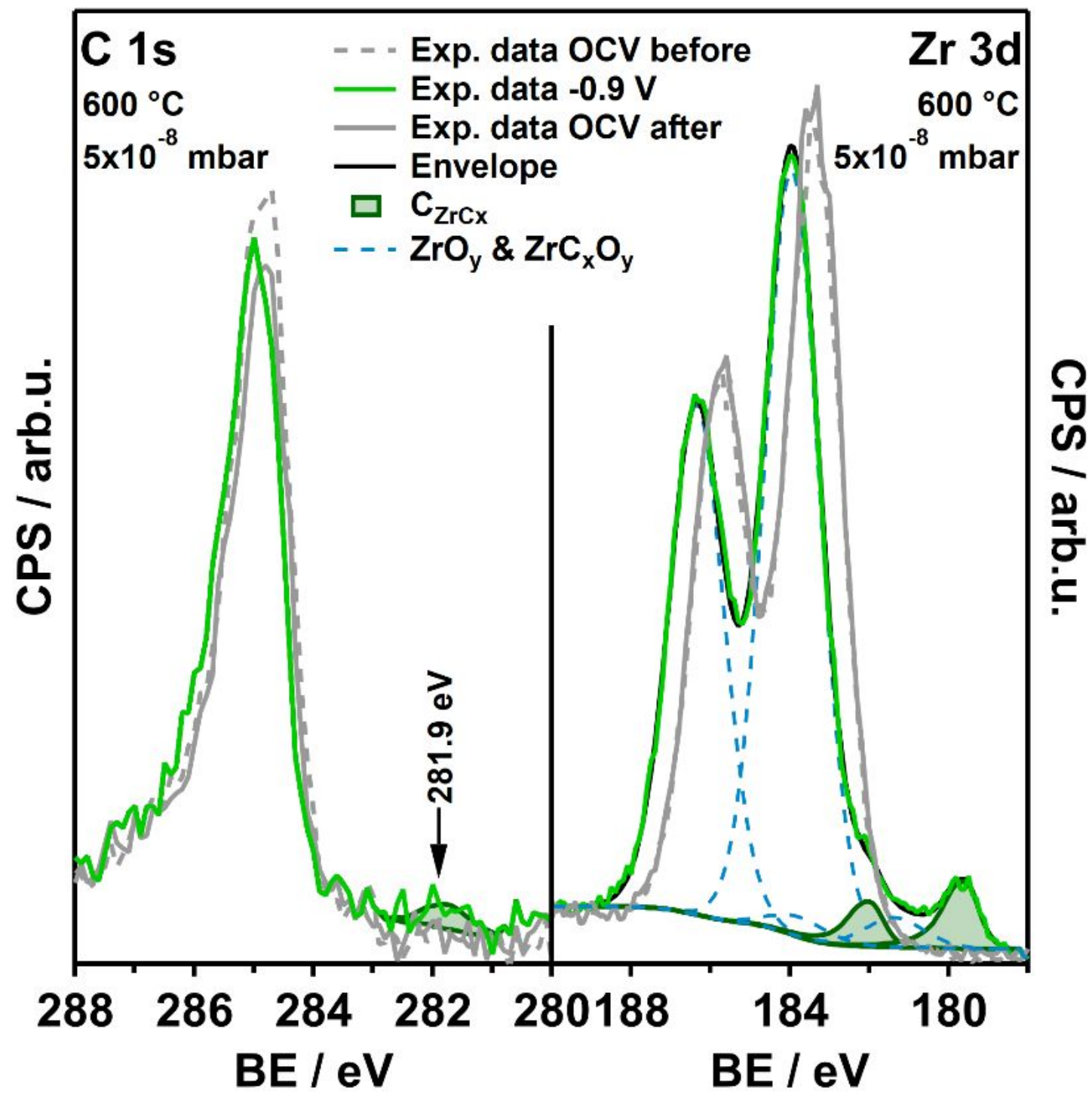

Figure S10. XP spectra series of the C 1s and Zr 3d regions recorded under vacuum conditions ($5{\cdot}10^{-8}$ mbar). The series is time-resolved (262 s between each spectrum) and starts with the C 1s and Zr 3d regions (gray dashed line) recorded under OCV conditions, followed by the detection of the same regions under cathodic polarization (green line, -0.9 V) and finally again under OCV conditions (solid gray line). The same fitting model as in Figure 3 and section S10 is applied to the experimental data. In essence, ZrO_y and ZrC_xO_y components (blue dashed line) as well as the C_{ZrCx} (green shaded) peak are fitted with a GL(30) fit function, whereas the ZrC_x fit utilizes a GL(30)T(1.3) asymmetric line shape. For the Zr 3d fit, the theoretical area ratio of the spin-orbit coupling ($d_{3/2}$ to $d_{5/2}$) was kept constant. The BE shift in the Zr 3d signal (gray vs. green lines) results from electrochemical polarization of the working electrode.

S12: Gas phase-dependent comparison of ZrC_x formation

Table S1 summarizes that the proportion of the ZrC_x component is a function of the CO partial pressure and is 6 at.% under HV, but 17 at.% in CO-containing atmosphere with p(CO) = 0.125 mbar (both measured on the same sample S3) and even more pronounced (up to 58 at.%) in 0.5 mbar CO. In this context, Table S1 further illustrates that the C_{ZrCx}/ZrC_x ratio ranges from 0.9 to 0.5 in agreement with the Zr-C phase diagram exhibiting a broad stability region of ZrC_x between $ZrC_{0.45}$ and $ZrC_{0.95}$[3].

Table S1. The table compares the ZrC_x formation under pure CO atmosphere (0.5 mbar) with H_2:CO atmosphere (p(CO) = 0.125 mbar) and with vacuum conditions. The fit results were derived from the experimental data of a not shown XP spectra series (H_2:CO) as well as from Figure S16 (H_2:CO) and S10 (vacuum). These experiments were conducted on the same sample (S3). Additionally, the table summarizes the ZrC_x formation in pure CO atmosphere (0.5 mbar), based on experimental data derived from Figure 3 (XP spectra series c) and a not shown XP spectra series. These results originate from two different samples (S2 and S4).

**Conditions**	**Sample**	**Set Voltage / V**	**ZrC_x / at.%**	**ZrO_y & ZrC_xO_y / at.%**	**ratio C_{ZrCx}/ZrC_x**
600 °C; H_2:CO = 3:1; 0.5 mbar p(CO) = 0.125 mbar (not shown)	S3	-0.90	16	84	0.9
600 °C; H_2:CO = 3:1; 0.5 mbar p(CO) = 0.125 mbar (Figure S16)	S3	-0.90	17	83	0.9
600 °C; $5\cdot10^{-8}$ mbar, HV (Figure S10)	S3	-0.90	6	94	0.7
600 °C; CO; 0.5 mbar (Figure 3, series c)	S2	-0.90	49	51	0.5
600 °C; CO; 0.5 mbar (not shown)	S4	-0.90	58	42	0.5

S13: Partially reversible carbon battery

Beside CO(g), the accumulated CO_2(g) and the main component in the residual gas of the NAP chamber, namely H_2O(g), are suggested to drive the reactions R2, R3 and R4 at the CE, equally leading to active transport of oxygen ions toward ZrC_x, and to the re-conversion of C_{ZrCx} to CO(g). As the TPB dimensions of the CE exceed that of the WE by far, the timescale to reduce equivalent amounts of CO(g) and residual CO_2(g) and/or H_2O(g) will be quite short, especially when considering the excellent exchange kinetics and the large surface area of the porous Pt/GDC cermet-based CE. In this respect, our cell may be considered as a "partially reversible carbon battery", featuring active gas-mediated carbon and electrolyte-mediated oxygen transfer between the electrodes.

S14: Detailed current characteristics of ZrC_x formation and decay

Figure S11 details the current characteristics during charging at the beginning of the carbon growth region g3 and discharging slightly past region g3 of Figure 2A. Panel A magnifies the discharge peak at the transition form -0.9 V to 0.0 V. The total charge transfer during discharge (Q=I x t) is compared to the preceding charging process and matches with the initial charging peak marked by dotted lines in Panels B and C, which is associated with the reduction of formally Zr^{4+} species and the generation of Zr^0 centers at the YSZ surface. The subsequent increase in current until 350 s (panel B) is tied to ongoing forward reaction R16 and enrichment of the reduced Zr centers by carbon. The diminished increase from 350 s onwards is interpreted to be the consequence of increasing ohmic electronic leak currents along the strongly reduced YSZ surface.

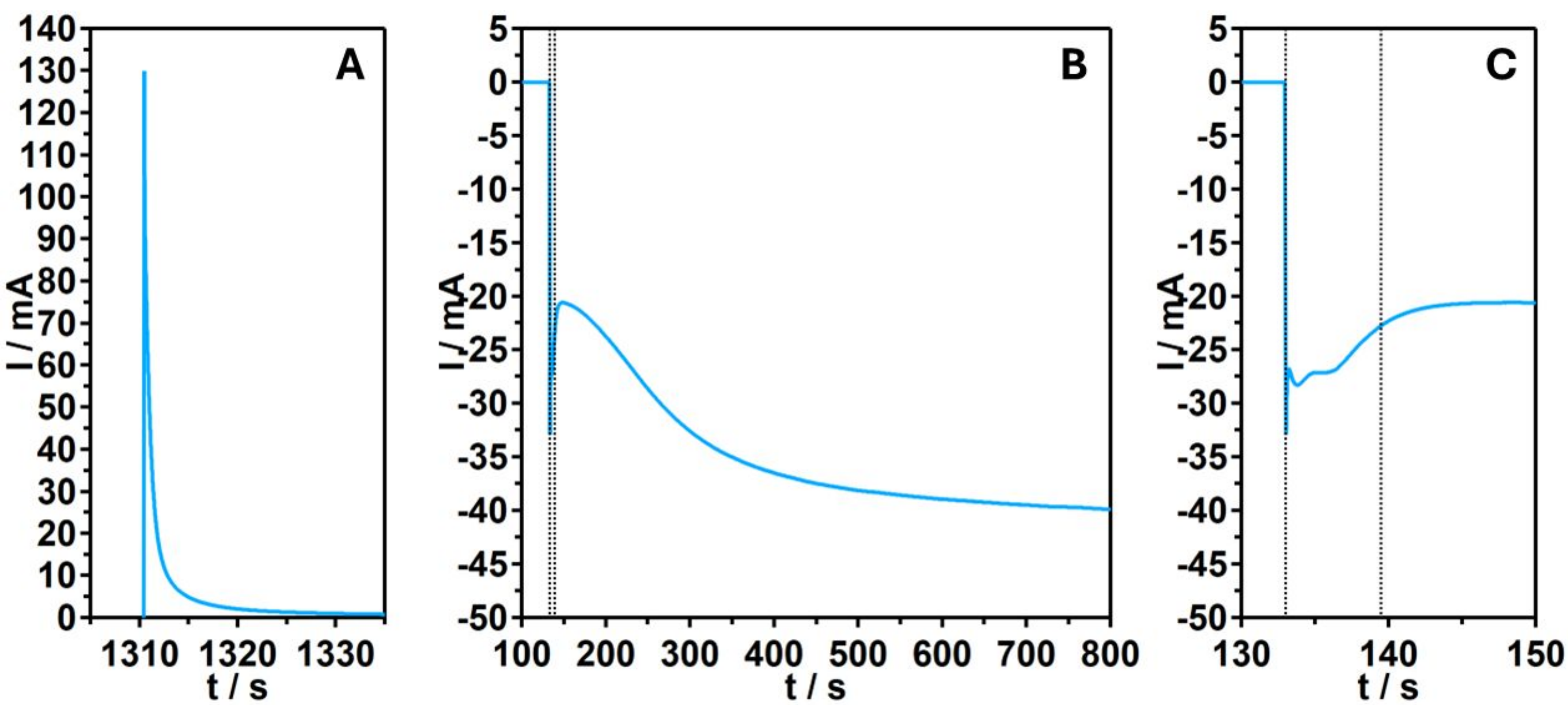

Figure S11. Time-resolved magnification of the current characteristics measured at the beginning of the carbon growth region g3 (panel B and C) and the transition during switching from -0.9V to 0.0 V (panel A) recorded at the very beginning of region d3 of Figure 2A.

S15: Detailed interpretation of final trends in Figure 3

As shown in Figure 3(e), the WE does not fully return to the status of (a) even after ≈900 s. Despite strongly reduced Ni 2p screening relative to (c) upon de-polarization, it maintains a lowered Ni 2p intensity relative to (a). Considering the constant Ni 2p signal after (d) and thus a rather complete carbon dissolution/decomposition process on Ni, and - following trace S2-d3 in Figure S8 - a steep decrease of the C_{TPB}/Zr ratio continued by a rather constant progression, we suggest the presence of two forms of carbon: one reactive form, suspected to originate from initially grown/spilled-over, and thus rather disordered carbon, and a less reactive form, most likely resulting from R15. We further suggest that the progressive conversion of disordered to ordered graphite-like carbon (via R15) gives rise to partially retarded reaction kinetics, i.e. $C_{sp3/sp2,\ disordered}$ is dissolved or reacts more quickly than kinetically stabilized, long range-ordered sp^2-like carbon e.g. in multilayered graphite domains[4]. If the reverse reaction of R14 is suppressed or only partially completed, a larger fraction of stable graphitic domains remains at the Ni surface, causing more Ni 2p shielding relative to (a). Nevertheless, the C 1s peak is clearly diminished from (d) to (e), while the Ni 2p shielding hardly changes. This contradicts ongoing carbon dissolution in Ni and/or electrochemical clean-off of C@Ni via the TPB, but agrees with a correlation between the Zr 3d and C_{TPB} integral peak areas observed during carbon growth with already extensive, but constant Ni 2p shielding on the same sample (Figure S12A, datapoints above 1000 s). This correlation (increasing C_{TPB}, decreasing Zr 3d and constant Ni 2p signals), interpreted as carbon overgrowing and thereby shielding the YSZ domains while maintaining the Ni 2p signal, shows strong similarities in terms of progression of the integral peak areas (d) to (e) of those signals. By comparing these integral peak areas, Figure S12B indicates that only in (e) the Zr 3d signal is fully reestablished, while in (d) it is still shielded by carbon, most likely from "redistributed" C_{TPB} after inverse spillover. We suggest that reverse reaction R9 is the reaction pathway driving the decomposition of this "redistributed" and therefore disordered C_{TPB} from (d) to (e), while re-establishing the ZrO_y and maintaining Ni 2p intensities.

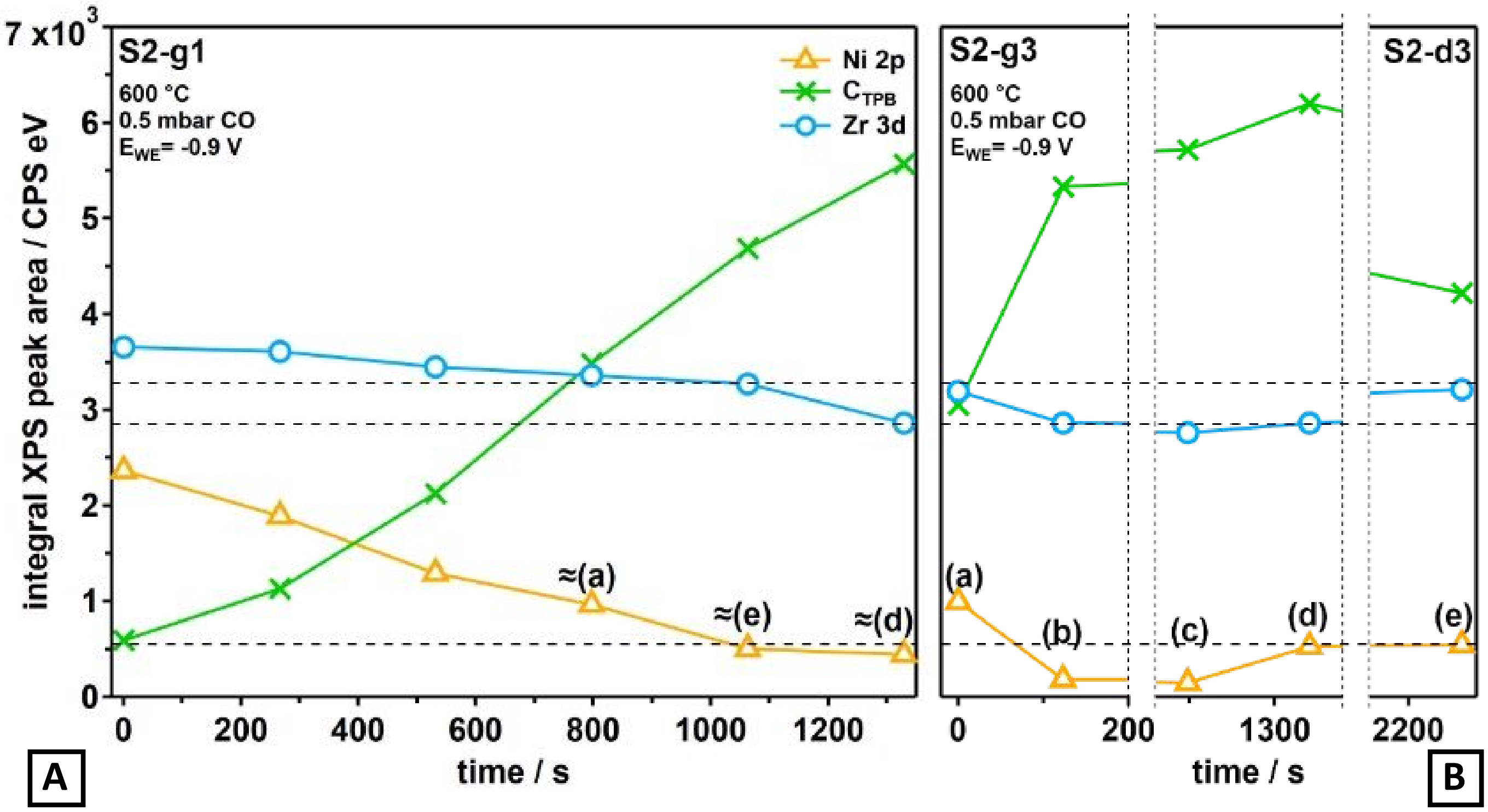

Figure S12. Panel A shows the integral peak areas of Ni 2p, C_{TPB} and Zr 3d signals during the carbon growth cycle g1 on sample S2. Panel B represents the integral peak areas from the ZrC_x formation cycle (g3) and subsequent dissolution/decomposition period (d3) on the same sample. The indices (a) to (e) highlight the stages of sample as used in Figure 2, 3, S8 and S9. The horizontal lines indicate similar peak areas for stage (a), (d) and (e) detected during carbon growth cycle g1.

S16: Energetics of carbon adsorption at Ni_4 and ZrO_2

To understand the formation of the ZrC_x layer, we use a smaller Ni_4 model, to be able to saturate the particle with carbon. Carbon binds relatively strongly at a Ni particle with ≈7.1 eV, much higher than filling one of the vacancies in the ZrO_2 caused by the Y doping, 3.6 eV. Ni particles are well known to form carbon nanotubes under similar conditions. Indeed, saturating Ni_4 with carbon does not lead to a significant drop in carbon binding energy, e.g. the 6th carbon still binds with 6 eV at Ni_4, forming mostly C_2 dimers (Figure S13). But carbon adsorption in the vacancy may also lead to CO formation with nearby lattice oxygen, which is favorable with -4.0 eV. The difference to the adsorption energy of carbon at Ni_4 remains notable, but the carbon adsorption energy at the ZrO_{2-x} rises to -4.9 eV, once the next atom is transferred to the surface. This means once an extra vacancy has been created, besides the "intrinsic" vacancy due to Y^{3+} doping, the transfer of carbon is solely about 1 eV uphill, which can be compensated experimentally by applying ≈0.9 V polarization. This rationalizes the energetics of a surface carbide species originating in the proximity of the Ni_4 particle (cf. data summarized in Table S2). Considering that more strongly reduced YSZ surfaces or even intermediate metallic Zr^0 sites can be generated experimentally, negligible reaction barriers for Zr carburization can be anticipated.

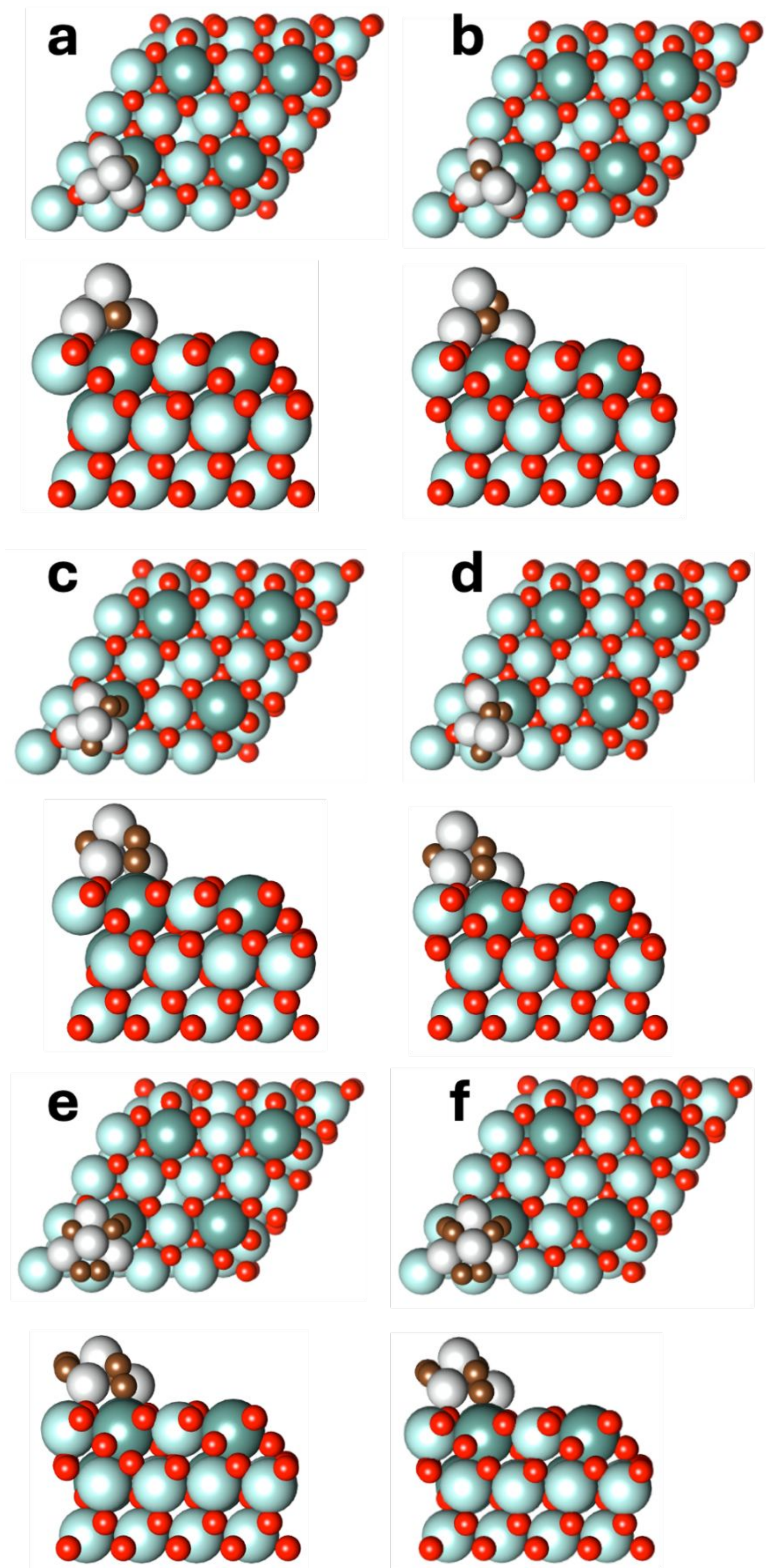

Figure S13. a) to f) Model systems with 1 through 6 carbon atoms attached to the Ni_4 particle.

Table S2. DFT-derived carbon adsorption energies on Ni_4 vs. ZrO_x

E_{ads} at a Ni_4 particle on ZrO_2(111): "Ni_4" or at the ZrO_2 support, "ZrO_2", with respect to the number of carbon atoms, #C with respect to a carbon atom in the gas phase as reference in eV/C. Adsorption at the support is followed by a desorption energy E_{des} of CO generated.

**Site**	**#C**	**E_{ads}**	**E_{des}**
Ni_4	1	-7.1	
	2	-6.9	
	3	-7.2	
	4	-6.2	
	5	-7.0	
	6	-5.9	
ZrO_2	1	-4.0	+0.6
	2[a]	-4.9	+1.3
	2	-5.4	+0.3
	3	-4.5	+1.1

[a] Alternative location of carbon atom

S17: Dissolution rate of C_{TPB} and reactivity of C_{TPB} with H_2

Figure S14 compares the dissolution kinetics of electrochemically grown graphitic C_{TPB} under high vacuum (HV) with those under H_2 atmosphere. By plotting the atomic ratio of C_{TPB}/Zr, the effect of adding H_2 on the dissolution of C_{TPB} into the Ni bulk was monitored as a function of time. The sigmoidal behavior allows to extract maximum decay rates (-d(C/Zr)/dt) at the respective inflection points (see section below Figure S14). The data reveal that the decay rates in 0.1 mbar H_2 atmosphere (blue traces) are ≈2.5 times lower than under HV conditions ($5 \cdot 10^{-8}$ mbar, orange trace).

Basically, the dissolution process of single atomic C_{ads} (sp^3-like) species resulting from thermal decomposition of graphitic C is possible at ≥600 °C on Ni surfaces[5], but, as suggested by Patera et al.[6], the partial hydrogenation of sp^2-bonded C at the edges of the decaying graphite layer (C_{edge}-H) rather stabilizes the edges and, therefore, suppresses the release of mobile single C_{ads} species from the graphite network, which is not a limiting factor under high vacuum conditions.

Our findings provide evidence for inhibited direct reactivity of C_{TPB} (largely consisting of C_{sp2}) with respect to process R7, associated with deactivation of the otherwise reactive graphitic C edges[6] with respect to carbon dissolution. Thus, among the potentially active carbon species to undergo R7, rather non-graphitic C@Ni states located at the surface are relevant, e.g. the initially formed sp^3-bonded C_{TPB} atoms resulting from the DFT-modelled CO activation mechanism at the Ni^0-YSZ phase boundary prior to their trapping in disordered sp^3/sp^2 networks and, eventually, ordered sp^2/graphite structure, as already suggested in[7].

To investigate a possible dissolution limitation due to carbon pre-saturated Ni subsurface and bulk regions, the H_2 clean-off experiment was repeated on the same, but carbon pre-saturated sample: S1-d2 (dark blue trace) showing a slightly lower dissolution rate (-d(C/Zr)/dt) compared to the pristine sample (S1-d1, light blue trace). Therefore, the dissolution rate was compared to a vacuum dissolution experiment carried out on a pristine sample (S2-d1, gray trace with circles). An even more decreased dissolution rate was observed on sample S1 while conducting a third dissolution experiment, but this time under HV (S1-d3, gray trace with triangles).

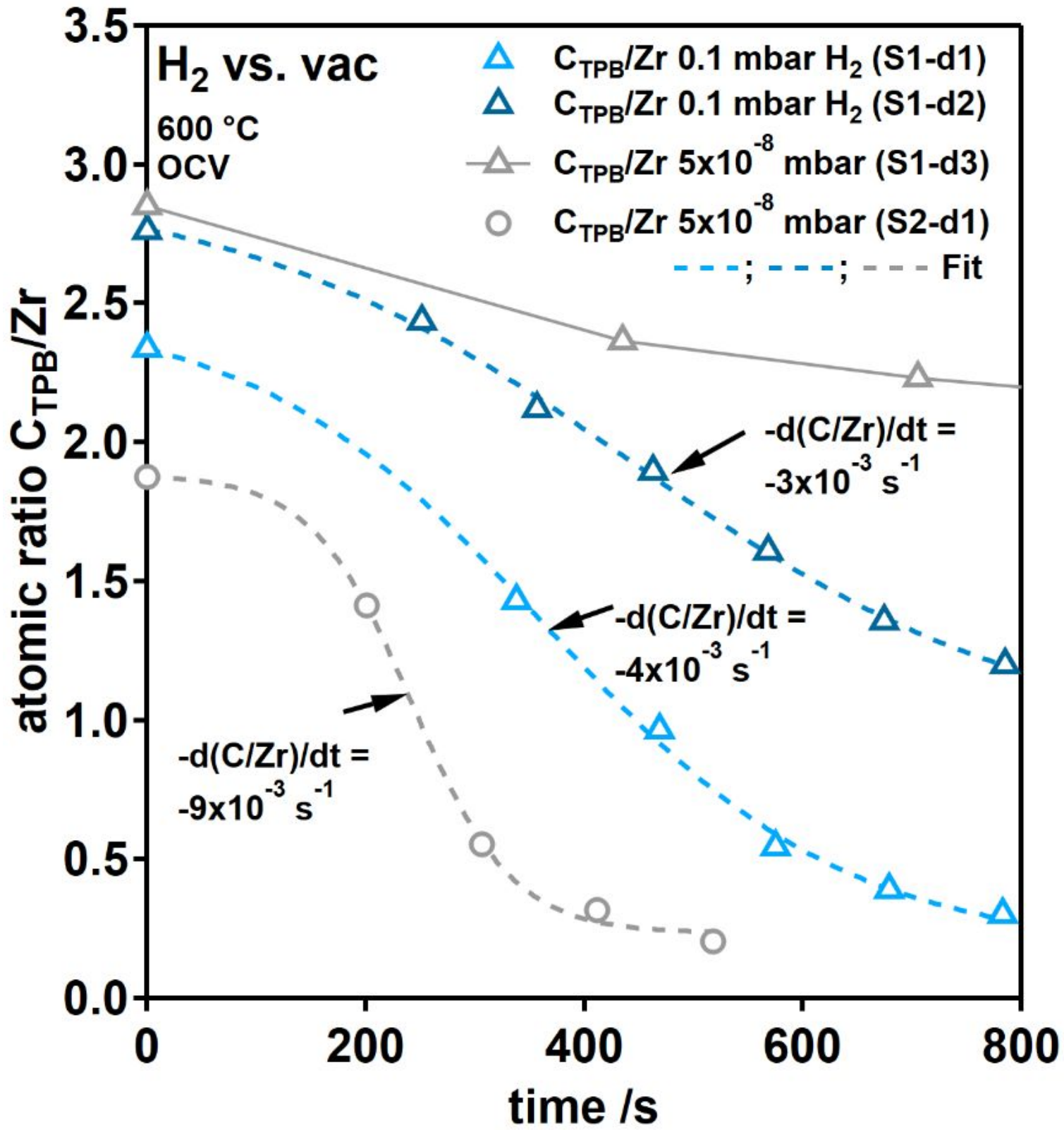

Figure S14. Decrease of the atomic ratio C_{TPB}/Zr plotted isothermally as a function of time at 600 °C. The gray data points correspond to the dissolution of C_{TPB} into the Ni bulk under high vacuum conditions ($5\cdot10^{-8}$ mbar). The blue data points correspond to the analogous experiments on the same sample (S1) in two different stages (pristine d1 and carbon pre-saturated d2) in 0.1 mbar H_2 atmosphere. Sigmoidal fit curves are plotted as dashed lines using the respective color, and maximum carbon loss rate numbers (-d(C/Zr)/dt) obtained at the marked inflection points are indicated.

Sigmoidal fitting approach (dashed curves): the obtained datapoints show a sigmoidal behavior, which can be interpreted as an interplay of geometrical and kinetic factors. In the beginning, multi-layer (bulky) carbon is detected only, meaning the inelastic mean free path (IMFP) of the photo electrons in $C_{graphite}$ is smaller than the thickness (d_C) of the carbon layer (IMPF < d_C). With time the carbon layer is depleted by either reaction R7 or metal-bulk dissolution which becomes spectroscopically increasingly fast when IMFP ≈ d_C and slows down when IMFP > d_C. Therefore, a fitting routine of the experimental data with equation (E1) appears as reasonable approach for data evaluation.

$$C/Zr(t) = C/Zr_{base} + \frac{C/Zr_{max}}{1+e^{\left(\frac{t_{half}-t}{rate}\right)}} \qquad \text{(E1)}$$

Differentiation of the resulting fit, allows us to determine the maximum rate (-d(C/Zr)/dt) at the inflection point of the sigmoidal curve. According to Figure S14, the -d(C/Zr)/dt rates obtained by tracking the C_{TPB} at 600 °C in H_2 atmosphere (light blue trace) vs. HV conditions ($5 \cdot 10^{--}$ mbar, gray trace with circles) differ roughly by a factor of ≈2.5. Meaning, when 0.1 mbar of H_2 is present in the gas atmosphere, the otherwise efficient HV dissolution of previously deposited C_{TPB} is prevented. Based on the superior H_2 adsorption kinetics of Ni and taking an atomic dissolution process of single atomic C_{ads} species into account, this results in the following interpretation: effective hydrogenation of C_{TPB} is leading to C_{ads}-H_x (x=2 to 3) species whenever those are separated from the C_{TPB} (mostly C_{sp2}) bulk, which tends to be rather insoluble. However, the further hydrogenation of C_{ads}-H_x (x=2 to 4) toward a CH_4 molecule (R7) is rather slow, and therefore C_{ads}-H_x species are poisoning the surface and decreasing the overall -d(C/Zr)/dt rate observed under H_2 atmosphere.

S18: Reactivity of polarization-induced C_{ZrCx} with H_2

Figure S15A shows the development of the C 1s region at 600 °C, starting with 0.5 mbar pure CO from 0 to 44 min at -0.9 V (C deposition), and followed by exposure to 0.125 mbar pure H_2 at OCV between 44 and 75 min (C dissolution and/or reaction). Thereafter, the gas phase was topped up to a 0.5 mbar H_2:CO=3:1 mixture and again biased to -0.9V. At first, this led to the re-establishment of a large C_{TPB} peak. After ~111 min, the polarization-induced ZrC_x species started to grow at ≈281.9 eV and increased during the next 13 minutes (inset). Figure S16, showing the response of the Zr 3d region to polarization with better resolution, provides evidence that a ZrC_x species is formed and persists solely under cathodic conditions (simultaneously recorded Zr $3d_{5/2}$ component at ≈179.5 eV).

When the voltage was turned off and OCV conditions were re-established after 124 min, the carbide peak vanished almost immediately (spectrum at 126 min). While keeping the electrode at OVC until 224 min, the carbide component did not re-appear, and the C_{TPB} again lost intensity due to C dissolution or clean-off reactions, despite the permanent presence of the syngas environment. By turning the -0.9 V voltage back on under otherwise identical conditions, the reappearance of the C_{ZrCx} peak was triggered (232 min), while the C_{TPB} intensity continued to grow in parallel. Changing the H_2:CO ratio to 2:1 (243 min) and to 1:1 (259 min), while maintaining -0.9V, showed little effect on the C_{ZrCx} and C_{TPB} intensities, except a minor decrease of the ratio. Most interestingly, both components even slightly increased over at least 11 min upon switching to 0.125 mbar pure H_2 at 298 min, indicating that (1) both species are rather unreactive toward H_2, and (2) that a certain reservoir of carbon has been built up in the preceding steps, which can be stabilized in a carbidic state under cathodic conditions. As soon as the voltage was switched back to OCV (after 309 min), the C_{ZrCx} peak vanished and C_{TPB} started to decrease again (spectrum at 311 min). Figure S15B depicts the integral C_{TPB} and C_{ZrCx} peak area plotted vs. time, focusing on the particular period between 111 and 311 minutes. The data confirm the potential-induced emergence of Zr carbide, which promptly disappears upon switching back to OCV. Simultaneously, C_{TPB} exhibits reproducible growth and dissolution/decomposition trends in response to cathodic polarization and gas phase composition. Considering that electrochemically grown C_{TPB}

covers predominantly the Ni surface (Figure S3), and that polarization-driven ZrC_x formation can be observed even under vacuum conditions ($5 \cdot 10^{-8}$ mbar; Figure S10), this result confirms the decomposition/spillover pathway of pre-deposited C_{TPB} (mainly consisting of C_{sp2}) on Ni^0 toward ZrC_x.

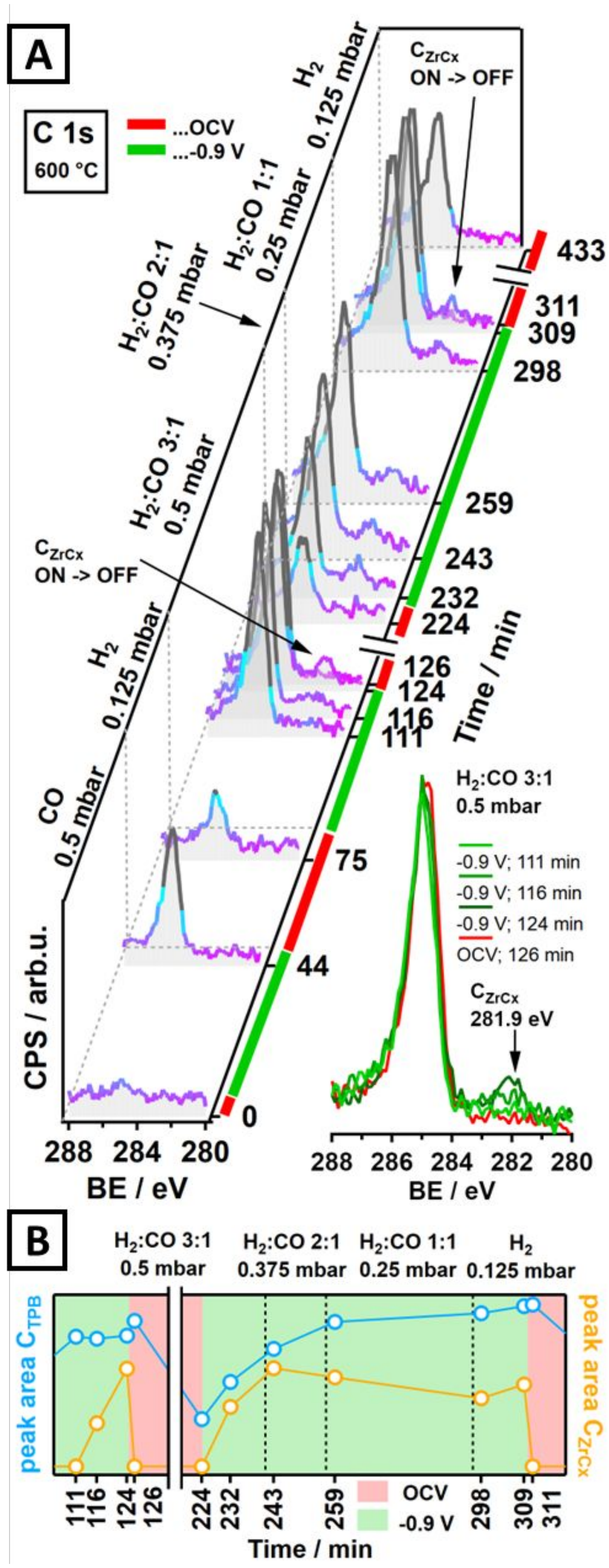

Figure S15. A: 3D-plot illustrating the chosen sequence of potential and gas phase conditions, alongside

selected C 1s spectra, vs. time. The inset highlights the growth of the C_{ZrCx} 1s component at ≈281.9 eV (-0.9 V polarization, 0.5 mbar H_2:CO=3:1, 600 °C), which promptly disappears at OCV. B: Development of integral peak intensities of the C_{TPB} and C_{ZrCx} components plotted vs. the chosen time sequence, revealing their relative growth and decay timescales, as well as their stabilities in variable gas compositions.

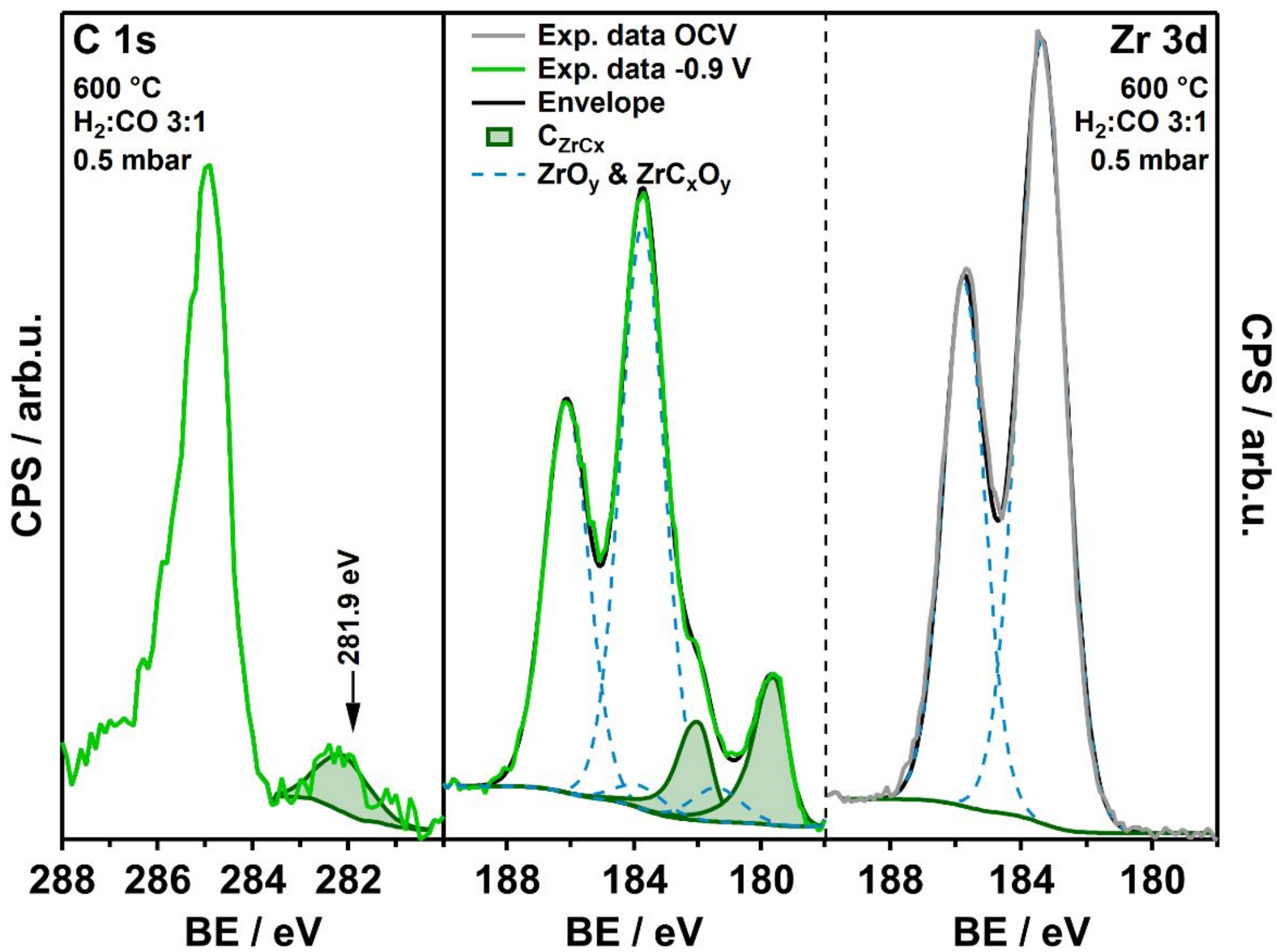

Figure S16. XP spectra of the C 1s and Zr 3d regions under -0.9 V cathodic polarization (green line) and the Zr 3d region under OCV conditions (gray line) as reference. The same fitting model as in Figure 3 and section S10 is applied to the experimental data. In essence, ZrO_y and ZrC_xO_y components (blue dashed line) as well as the C_{ZrCx} (green shaded) peak are fitted with a GL(30) fit function, whereas the ZrC_x fit utilizes a GL(30)T(1.3) asymmetric line shape. For the Zr 3d fit, the theoretical area ratio of the spin-orbit coupling ($d_{3/2}$ to $d_{5/2}$) was kept constant.

References

(1) Biesinger, M. C. Accessing the robustness of adventitious carbon for charge referencing (correction) purposes in XPS analysis: Insights from a multi-user facility data review. *Appl. Surf. Sci.* **2022**, *597*, 153681. DOI: https://doi.org/10.1016/j.apsusc.2022.153681.

(2) Hauser, D.; Grießer, C.; Wernig, E.-M.; Götsch, T.; Bernardi, J.; Kunze-Liebhäuser, J.; Penner, S. The pervasive presence of oxygen in ZrC. *Surfaces and Interfaces* **2022**, *34*, 102373. DOI: https://doi.org/10.1016/j.surfin.2022.102373.

(3) Hugosson, H. W.; Eriksson, O.; Jansson, U.; Johansson, B. Phase stabilities and homogeneity ranges in 4d-transition-metal carbides: A theoretical study. *Physical Review B* **2001**, *63* (13), 134108. DOI: https://doi.org/10.1103/PhysRevB.63.134108.

(4) Bayer, B. C.; Bosworth, D. A.; Michaelis, F. B.; Blume, R.; Habler, G.; Abart, R.; Weatherup, R. S.; Kidambi, P. R.; Baumberg, J. J.; Knop-Gericke, A.; et al. In Situ Observations of Phase Transitions in Metastable Nickel (Carbide)/Carbon Nanocomposites. *J. Phys. Chem. C* **2016**, *120* (39), 22571-22584. DOI: https://doi.org/10.1021/acs.jpcc.6b01555.

(5) Dahal, A.; Batzill, M. Graphene–nickel interfaces: a review. *Nanoscale* **2014**, *6* (5), 2548-2562, 10.1039/C3NR05279F. DOI: https://doi.org/10.1039/C3NR05279F.

(6) Patera, L. L.; Bianchini, F.; Troiano, G.; Dri, C.; Cepek, C.; Peressi, M.; Africh, C.; Comelli, G. Temperature-Driven Changes of the Graphene Edge Structure on Ni(111): Substrate vs Hydrogen Passivation. *Nano Lett.* **2015**, *15* (1), 56-62. DOI: https://doi.org/10.1021/nl5026985.

(7) Thurner, C. W.; Haug, L.; Winkler, D.; Griesser, C.; Leitner, M.; Moser, T.; Werner, D.; Thaler, M.; Scheibel, L. A.; Götsch, T.; et al. Electrocatalytic Enhancement of CO Methanation at the Metal-Electrolyte Interface Studied Using In Situ X-ray Photoelectron Spectroscopy. *C* **2023**, *9* (4), 106. DOI: https://doi.org/10.3390/c9040106.
